# Genomic solutions to metadata challenges: a case study on the 1000 Bull Genomes Project

**DOI:** 10.1186/s12711-026-01061-w

**Published:** 2026-07-24

**Authors:** Johanna-Sophie Schlüter-Bartram, Felix Heinrich, Mehmet Gültas, Armin O. Schmitt

**Affiliations:** 1 Breeding Informatics Group, Department of Animal Sciences, University of Göttingen, Margarethe-von-Wrangell-Weg 7, 37075 Göttingen, Germany; 2 Center for Integrated Breeding Research (CiBreed), Büsgenweg 5, 37077 Göttingen, Germany; 3 Faculty of Agriculture, South Westphalia University of Applied Sciences, Lübecker Ring 2, 59494 Soest, Germany

## Abstract

**Background:**

Advancements in sequencing technologies have led to an unprecedented availability of whole-genome sequencing data in all life sciences, including livestock research. However, this raises concerns regarding the accuracy of the associated metadata, particularly information on an individual’s subspecies or breed.

**Results:**

In this analysis, the 1000 Bull Genomes project was used as an example for a large-scale dataset with structured metadata. We applied a framework combining a query of the NCBI BioSamples database with principal component analysis, admixture analysis, and distance metrics based on the genomic information to assess metadata integrity. The main decrease in metadata quality results from missing breed assignments for 6% (n= 360) of the samples. Moreover, we identified 3% (n= 183) subspecies misassignments and 26 % (n= 1635) of the individuals are involved in the correction of breed assignments. In total, 603 animals receive metadata corrections of the breed or subspecies.

**Conclusion:**

Our study highlights the necessity of rigorous metadata assessments, even when utilizing well-established datasets. Furthermore, we emphasize the importance of providing comprehensive and accurate metadata when depositing genomic information in public databases to ensure the integrity and utility of such datasets.

**Supplementary Information:**

The online version contains supplementary material available at 10.1186/s12711-026-01061-w.

## Background

Globally, cattle are one of the most important livestock species, providing milk, beef, draught power and other resources. The domestication of the current *Bos* species occurred in two separate events in the Fertile Crescent and the Indus Valley leading to the formation of two subspecies: *Bos taurus taurus* and *Bos taurus indicus* [1, 2]. The humpless taurine cattle breeds are most prevalent in Europe, northern parts of Asia, as well as western and central Africa [3, 4]. Indicine cattle carry a hump and are predominantly found in the southern parts of Asia, as well as northeastern and western Africa [3, 4]. Breeds with mixed ancestry of different proportions, such as the African Sanga and Zenga [5], are found in the central and southern regions of the African continent [4] and are valued for their improved adaptation in several regions [6].

The past decade has seen a rapid rise in studies on whole genome sequencing (WGS). In this process, consortia provided valuable large datasets in agricultural sciences, such as the 1K Chicken Genomes Project [7], the VarGoats project [8], and the 1000 Bull Genomes Project [9].

The 1000 Bull Genomes Project [9] is one of the most comprehensive collection efforts for genomic information in livestock sciences, comprising data for both *B. taurus* and *B. indicus* individuals, and mixed forms. The whole genome sequencing is processed through a standardized pipeline but submitted by a diverse group of contributors. In the past, the project’s dataset has frequently been employed as a resource for imputations (e.g. [10]), but also in analyses of population structures and diversity assessments in *B. taurus* breeds [11, 12]. The project evolved in several cycles, from 133 individuals of two breeds to several thousand animals representing the global diversity of cattle [9], enabling the analysis of big genomic data in agricultural research at an unprecedented scale. A cornerstone to successfully process and reuse these large amounts of data, with respect to Wilkinsons’ FAIR principles (findability, accessibility, interoperability, and reusability) [13], is the availability of extensive high-quality metadata [14–16].

Contrary to the genomic information, not the size, but format variability, and completeness are the challenges associated with metadata quality [14, 15]. Previously, the need for metadata standards and reuse opportunities in agricultural sciences have been addressed by the FAANG project [17] and the AgBioData consortium [16]. While these efforts promote the provision of meaningful metadata, the usage of these approaches to detect errors in already existing metadata is limited. In the case of livestock sciences, metadata errors may include missing data, misassignments (e.g., wrong or meaningless subspecies assignments), or inconsistent naming of breeds. The simultaneous growth of big datasets and difficulty in detecting metadata errors [15] presents a clear challenge. Given this, it is arguable that metadata–not genomic data– will become critical in big datasets, underpinning the urgency of finding efficient approaches to detect existing metadata errors at scale.

The consequences of misassignments at the level of metadata for genomic datasets range from a reduction in sample size, which may limit the applicability of statistical tests, to changes in the results of analyses when data are split according to erroneous variables. In contrast to missing information, misassignments are difficult to detect [15]. One illustrative example to highlight the permanent consequences that incorrect assignments in metadata can have: when genomic data are used to train a classifier to predict breeds, lineages or subspecies as it has been done in several studies in the past [18–20], a ground truth of the classes to predict is required for training such a model and estimating its performance. In this case, errors in the original metadata could affect the quality of a classifier without raising any error messages.

Given the impact of the metadata on the (re)usage of information, a need for a systematic assessment method to detect ambiguities in large-scale, consortia generated datasets arises, for which we propose utilizing the associated genomic information. Using the 1000 Bulls Genomes Project as an example, this study aims to develop and apply a scalable genomic framework to curate metadata misassignments, at both breed and subspecies level, in large-scale genomic datasets.

## Methods

### Dataset description and preparation

The 1000 Bull Genomes Project is a unique collection of large-scale genotypes offering an optimal opportunity to use genome based information to assess the current genetic variation in *B. taurus* and *B. indicus* cattle. We analyse the metadata and genomic information from the current run (Run 9) [9], which consists of data from 6191 individuals, with genomic information recorded for more than 115 million genomic positions. The metadata for each animal includes records on the subspecies, breed, and individual identifier (ID) along with quality measures and further information. The metadata forms three levels of a hierarchical structure, assigning individuals to breeds and breeds to subspecies.

**Table 1:** Summary of subspecies, number of individuals, and breeds. The term "unknown" is used in the metadata for individuals with a known subspecies but no information regarding the breed is given

Subspecies	Number of individuals	Number of breeds	
*B. taurus*	5204	189	+ unknown
*B. indicus*	606	43	+ unknown
*B. taurus* x *B. indicus*	288	21	
Other small groups	93	13	+ unknown
Sum	6191	267	

The majority of the individuals in the dataset belong to the *B. taurus* subspecies and about 10 % are *B. indicus* individuals, followed by admixed individuals and small groups (see Table 1). Similarly, the number of breeds recorded for taurine individuals exceeds that for indicine individuals (see Table 1). However, not all individuals carry a meaningful subspecies or breed assignment.

As members of the 1000 Bull Genomes Project, we received non-imputed variant calling files for all samples, which were filtered to a minimum quality score of 40 (QUAL), before imputing the genomic information with Beagle (v.5.4, [21]), as described by Heinrich et al. 2025. [22], and converted to binary files with *PLINK* (v.1.9, [23]) for compression. For the following analysis, the *R* program (v.3.6.3, [24]) and the *BGData* package (v.2.4.1, [25]) was used for memory mapping to access the genomic information. As we aim to demonstrate how to work with existing datasets, and since re-sequencing samples of low coverage is beyond the scope of this study, we decided to retain all individuals in the dataset, despite the variations in coverage. To estimate the effect of more stringent filtering, we exemplarily filtered chromosome 29 additionally for an individual coverage of at least 10 reads using BCFtools [26], prior to imputation.

### Analysis framework

Our framework analyses the subspecies and breeds from the 1000 Bull Genomes Project. Consequently, misassignment errors may arise at both levels of this hierarchy and due to different error sources, which makes it necessary to use various methodological approaches to identify them. For the explorative analysis of metadata assignments in large genomic datasets with hierarchical structures in the metadata, we propose a framework that contains five steps:

**Step 1: Recovery of meta-information** To reduce the amount of missing information the NCBI BioSample database [27] was queried for samples with available identifiers by using *rentrez* (v.1.2.3, [28]), to retrieve breed and sex information for all samples with corresponding references. For samples with no breed assignment in the original metadata, the breed assignments from the NCBI were used to replace the term "unknown". This step was omitted for samples lacking a BioSample identifier.

**Step 2: Detection of subspecies’ misassignments** We conducted a principal component analysis (PCA) using 50,000 randomly sampled genomic positions across all autosomes, without consideration of even spacing, as a sufficiently large number to obtain consistent results while maintaining memory efficiency. The PCA was conducted in R with the prcomp function. In order to identify breeds that are particularly distant from their assigned subspecies, the first two principal components are visualized using the *ggplot2* library (v.3.5.1, [29]). We classify misassignments of subspecies into three groups: (1) a switch between the subspecies *B. taurus* and *B. indicus*, (2) a switch between the subspecies and hybrids, or (3) the assignment of multiple subspecies to one breed. Additionally, ambiguous and missing subspecies information, which actually exists in linked databases, are considered to be misassignments.

**Step 3: Investigation of potentially misassigned subspecies** Breeds that are found to be substantially separated from their assigned subspecies in the PCA plot undergo a literature search to determine whether or not the assigned subspecies are consistent with previous (genomic) analyses. This procedure also applies to the uniformity of the assigned subspecies for a breed, which enables the identification of single misassigned individuals.

In our analysis, similar to previous studies, PC1 was found to reflect the differentiation between taurine and indicine cattle [20, 30–33]. To validate the PCA separation an admixture analysis was performed. Therefore, PC1 was divided into ten equal sections before randomly sampling an equal number of individuals (69 individuals per section), resulting in a total of 690 individuals across all sections. The sample size was determined by the minimum number of individuals available within the different sections. The right outermost individuals were limited to indicine and left outermost individuals were limited to taurine individuals, based on the assigned subspecies. Next, the genomic information across all autosomes for these individuals was further filtered to non-monomorphic sites (minor allele count $>=$ 1) using *PLINK*.

An analysis of the population structure for the present admixture among these individuals is then performed with *SCOPE* [34] in unsupervised mode. Here, the number of ancestral populations (k) is set to 2, as we assume the presence of two main populations: *Bos taurus* and *Bos indicus*. The sampling and admixture analysis was repeated five times to ensure a better representation of all populations, even in sections with a high density of individuals. We decided against a minor allele frequency filter to avoid the loss of globally rare alleles that may be common in smaller populations and used all available variants. Beyond this general assessment, we also used SCOPE to analyse the ancestral populations for breeds with missing subspecies assignments, adding 100 individuals from the left and right outermost sections of PC1, to ensure the detection of the subspecies ancestry and no other effects.

Instead of setting arbitrary thresholds for admixture proportions for taurine, mixed and indicine individuals, we used the SCOPE results from the sampling across PC1 to determine thresholds for the subspecies. We calculated the average observed admixture at the first and third quartile of PC1 positions across the five repetitions. Consequently, the thresholds reflect a PC1 position and average admixture simultaneously.

**Step 4: Detecting breed misassignments** To identify potentially misassigned individuals or groups at the level of breeds, we distinguish between three possible cases: (1) one breed is saved under two names, (2) two breeds are saved under one name and (3) individuals with a potentially known breed are labeled "unknown" or "other", i. e. they are considered not to belong to their actual breed.

The erroneous assignment of two breeds, where one is assigned incorrectly, may further stem from different error sources:

1. *Typographical errors in breed names*: These errors can arise from misspellings or other typing discrepancies, such as missing letters or letter transpositions.
2. *Variations of breed name components*: Parts of the breed name are added, modified, or transposed in one group. Detecting such errors requires expert knowledge of the conventions of breed nomenclatures and existing breeds.
3. *Breed names in different languages*: One breed may be assigned different names in different languages and dialects or have multiple names in one language. Identifying these errors requires linguistic expertise, demanding expert knowledge for each breed under consideration.

Following the outline above, many error sources cannot be detected automatically due to the requirements of expert knowledge (e.g. in several languages), which poses considerable hurdles to resolving metadata misassignments using the metadata alone. Considering this barrier, we propose using the genetic data to resolve such ambiguities.

**Step 5: Investigation of potentially misassigned breeds** Breeds with highly similar names are visualized on the (previously constructed) PCA plot. Those that are displayed in close proximity in the PCA are subjected to further analysis steps. We calculated the genomic distances (1-IBS) of all individuals with *PLINK*, using all available genomic positions. The distance of individuals from the breeds in question are examined as heatmaps and trees, constructed using UPGMA. Given the breeds appear as similar, they are submitted to an analysis of their ancestral population admixture using *SCOPE*. *SCOPE* was run in unsupervised mode, on all available variants, letting the number of assumed ancestral populations (k) vary between 2 and the number of groups +1 to further validate the observations made in the PCA.

Additionally, the pairs are investigated based on previous analyses regarding their given names. If this already results in the identification of a misassignment due to typing errors or a different name in the original publication and the metadata in the 1000 Bull Genomes Project, the pairs are grouped based on this information.

Further, we assessed the sex of individuals using non-imputed Y-chromosome data by examining the extent of missing data and incorporated the sex information obtained in Step 1 of the framework where available.

## Results

In this case study, we present an analysis framework for genomic information to identify misassignments in the associated metadata in five steps. To work as efficiently as possible, techniques to reduce storage and memory requirements through file compression, memory mapping and sampling are employed.

Splitting the dataset by subspecies and counting the breeds within them results in 267 breeds (see Table 1), while querying the total number of breeds results in 262 breeds. These results are not compatible, because several breeds are assigned to more than one species. An investigation of possible misclassifications is required to avoid possible mistakes in further analysis. Before investigating the breeds, we minimize the amount of missing information.

**Table 2:** Table of cattle whose breed was reported as ’unknown’ by the submitters in the metadata and the breeds identified by query of NCBI for the same samples

No	Breed	n	Consequence	BioProject	References
1	Asturiana	13	Additional breed	PRJEB38981	[46]
2	Bagaria	10	Additional breed	PRJNA698721	[47]
3	Bale	10	Additional breed	PRJNA698721	[47]
4	Baoule	7	Additional breed	PRJEB39924	[48]
5	BohaiBlack	2	Additional individuals	PRJNA379859	[41]
6	Brahman	31	Additional individuals	PRJNA555741	[49]
7	British White	1	Additional breed	PRJEB35299	[50]
8	Butana	2	Additional individuals	PRJNA574857	[51]
9	Deoni	3	Additional breed	PRJNA699427	
10	Djakkore	7	Additional breed	PRJEB39924	[48]
11	Gloucester	2	Additional breed	PRJEB35299	[50]
12	Gobra	6	Additional breed	PRJEB39924	[48]
13	Gourounsi	3	Additional breed	PRJEB39924	[48]
14	Gudali	1	Additional breed	PRJNA596606	[52]
15	Hereford	20	Additional individuals	PRJNA663547	[53]
16	Holstein	7	Additional individuals	PRJNA526664, PRJNA667172	[54, 55]
17	Holstein mix	1	Additional breed	PRJNA625124	[56]
18	Irish moiled	1	Additional breed	PRJEB35299	[50]
19	Japanese Black	11	Additional breed	PRJNA283480	[35]
20	Kapsiki	1	Additional breed	PRJNA596606	[52]
21	Kenana	4	Additional individuals	PRJNA574857	[51]
22	Leiqiong	2	Additional individuals	PRJNA283480	[35]
23	Limousin	5	Additional individuals	PRJNA526664	[57]
24	Luxi	1	Additional individual	PRJNA283480	[35]
25	Maure	4	Additional breed	PRJEB39924	[48]
26	Mongolian	16	Additional individuals	PRJNA598339	[58]
27	Mututu	4	Additional breed	PRJNA604048	[42]
28	Namchi	1	Additional breed	PRJNA596606	[52]
29	Nanyang	2	Additional individuals	PRJNA283480	[35]
30	Ndama	4	Additional breed	PRJEB39924, PRJNA604048	[42, 48]
31	NDama	4	Additional individuals	PRJEB39330, PRJNA574857	[51]
32	Pustertaler Sprinzen	1	Additional breed	PRJEB35299	[50]
33	Qinchuan	37	Additional individuals	PRJNA283480	[35]
34	Red Angus	18	Additional individuals	PRJNA283480	[35]
35	Red Fulani	1	Additional breed	PRJNA596606	[52]
36	Semien	10	Additional breed	PRJNA698721	[47]
37	Simmental	1	Additional individuals	PRJNA677947	[59]
38	White Fulani	1	Additional breed	PRJNA596606	[52]
39	Yanbian	2	Additional individuals	PRJNA283480	[35]
40	Yunnan	2	Additional breed	PRJNA283480	[35]
		Sum 259			

### Metadata recovery

Initially, breed information is missing for about 6% (360 animals) of the individuals in the metadata. To mitigate this problem to some extent, the information was retrieved from entries in the NCBI database by their BioSample identifiers. The proportion of missing breed assignments was reduced to 2% of animals, leaving only 101 animals without a breed assignment (see Table 6). The identification of 24 additional breeds (see Table 2) increased the number of breeds to 286.

Furthermore, the sample sizes for 16 breeds already represented in the original metadata were increased by including additional individuals. The breed information was recovered for all indicine cattle, providing a clearly improved resolution for the smaller subspecies in the dataset. The main challenge associated with this step is the heterogeneous recording of the breed in the NCBI records: in some samples the breed information was stored in the "title" e.g. the breed Asturiana (PRJNA698721), while in other cases the breed was stored under "infraspecies" or had to be retrieved from the supplementary files of associated publications.

Moreover, in a particularly challenging case (BioProject PRJNA283480) the breed information was not directly provided by the NCBI, but by supplementary files of the original publication [35]. Some of the identifiers provided for the samples by the NCBI and the metadata of their publication did not fit completely, due to the replacement of a single letter in some samples of the breed Qinchuan. We treated this as a typing error, as the number of the samples and rest of the identifiers matched. The identification of the breed samples required substantial effort due to missing breed labels and identifier errors, which made the samples less findable and reduced the overall metadata quality. Beyond misassignments in the metadata, breed recovery from NCBI itself may also cause the erroneous detection of two breeds where only one exists (see section below), as the alternative spelling of NDama as Ndama results in the interpretation as two breeds when working with case-sensitive software. In this case the spelling from the original metadata was adapted.

**Fig. 1:**
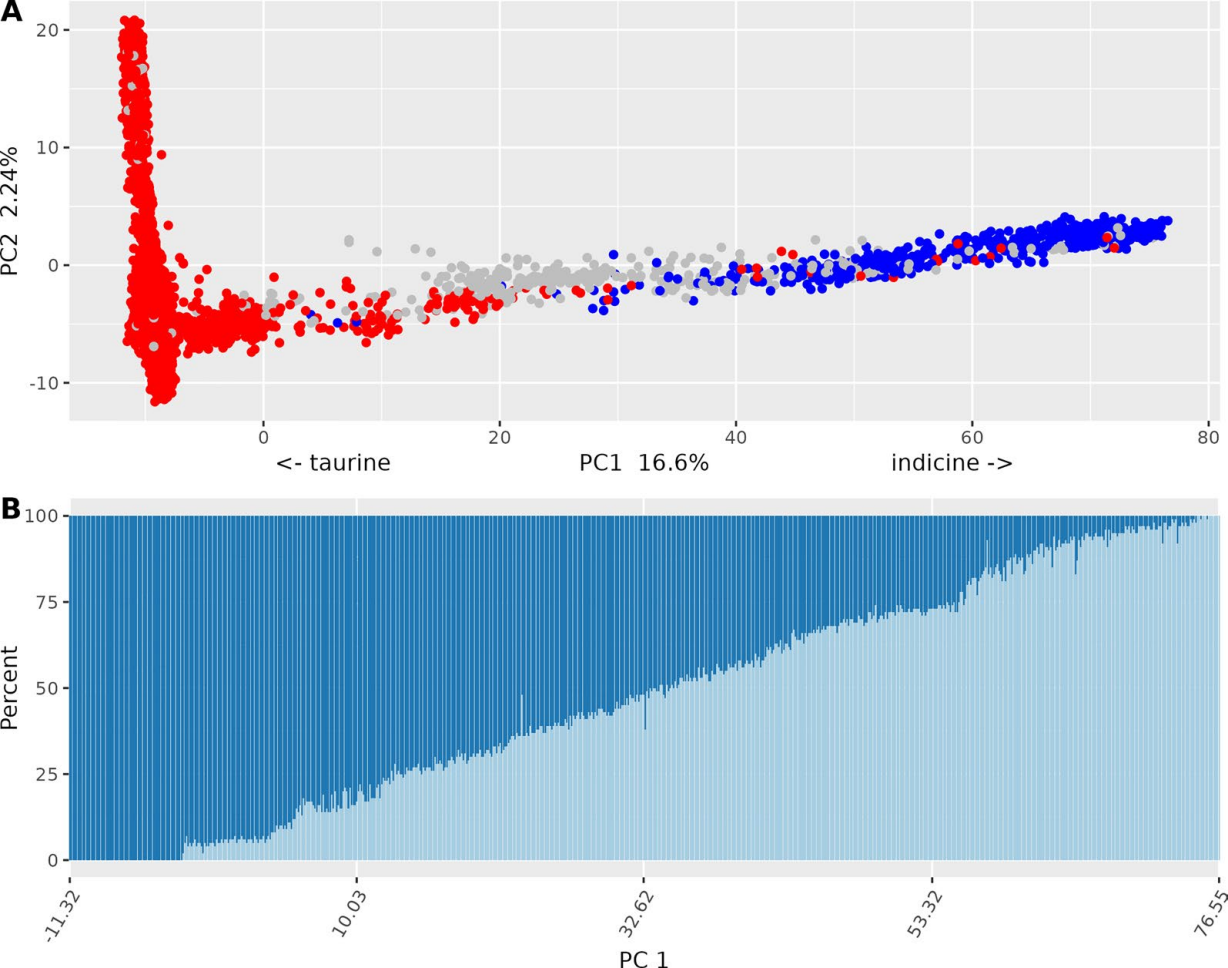
**A** PCA of all individuals (n= 6191) conducted on 50k randomly selected genomic positions, colored by the submitter-assigned subspecies, highlighting *B. taurus* (red) and *B. indicus* (blue), while all remaining subspecies groups are displayed in grey. **B** Results of the analysis of ancestral populations run on 690 individuals, evenly sampled in 10 sections across PC1, ordered by their respective position on PC1, displaying the populations relating to *B. taurus* (blue) and *B. indicus* (light blue)

### Subspecies level misassignments

To identify misassignments, we first performed a PCA. The results of the PCA show a continuous scale on PC1 (see Fig. 1 A). The polar ends of this continuous scale are formed by European *B. taurus* breeds on the left and *B. indicus* breeds on the right. These ends also correspond to the areas with the highest densities of observations, while the sections in between have notably fewer observations. When colouring the observations according to the subspecies assigned by the submitters, several inconsistencies are apparent at first glance, as individuals assigned to the subspecies *B. taurus* are displayed in the cluster of *B. indicus* individuals and vice versa. Across PC2 the taurine cattle are spread out, with the Holstein cattle clustered at the upper end of PC2 and the beef or dual purpose breeds clustered more at the lower end of PC2. Unlike taurine breeds, which exhibit a consistent trend, indicine and hybrid breeds, such as the Brahman and Nelore present unique characteristics that introduce specific obstacles in aligning with this pattern. Furthermore, the general distribution of admixed and indicine animals across PC2 does not show a separation as in the taurine individuals.

Analysing the ancestral populations of the individuals sampled evenly across PC1 (see Supplementary Figure S20) by setting k=2, a continuous gradient between the two main subspecies *B. taurus* and *B. indicus* is clearly visible when arranging the x-axis based on the PC1 values (see Fig. 1 B ). In agreement with previous findings that indicine admixture is rarely observed in European taurine breeds [6], few samples in the taurine cluster display mixed ancestry when setting k=2. On the contrary, taurine ancestry is more common in indicine cattle, which may be due to the smaller proportion of indicine cattle in the 1000 Bull Genomes Project (see Fig. 1). Furthermore, the results of the ancestral population analysis performed on all available genomic positions mirror the results of the PCA performed on 50k randomly sampled genomic positions across all autosomes, which is a small fraction of the input to *SCOPE*. Given this observation, it can be seen that a relatively small sample, including a random selection of input variables, yields results comparable to a high throughput analysis. The PCA displays a highly similar result, across repetitions and increased marker numbers (see Supplementary Figure S19). Further, no systematic deviation is observed between the data with additional filtering for coverage and the non-filtered data (see Supplementary Figure S21). Regarding the challenge of assigning cattle to distinct subspecies, our exploratory approach highlights the difficulty of establishing hard boundaries and assigning breeds of admixed populations to one of the subspecies. The continuous nature of the admixture leads to many breeds to be considered hybrids with a certain degree of "taurinicity" or "indicinicity", depending on the admixture proportions that are shared.

**Fig. 2:**
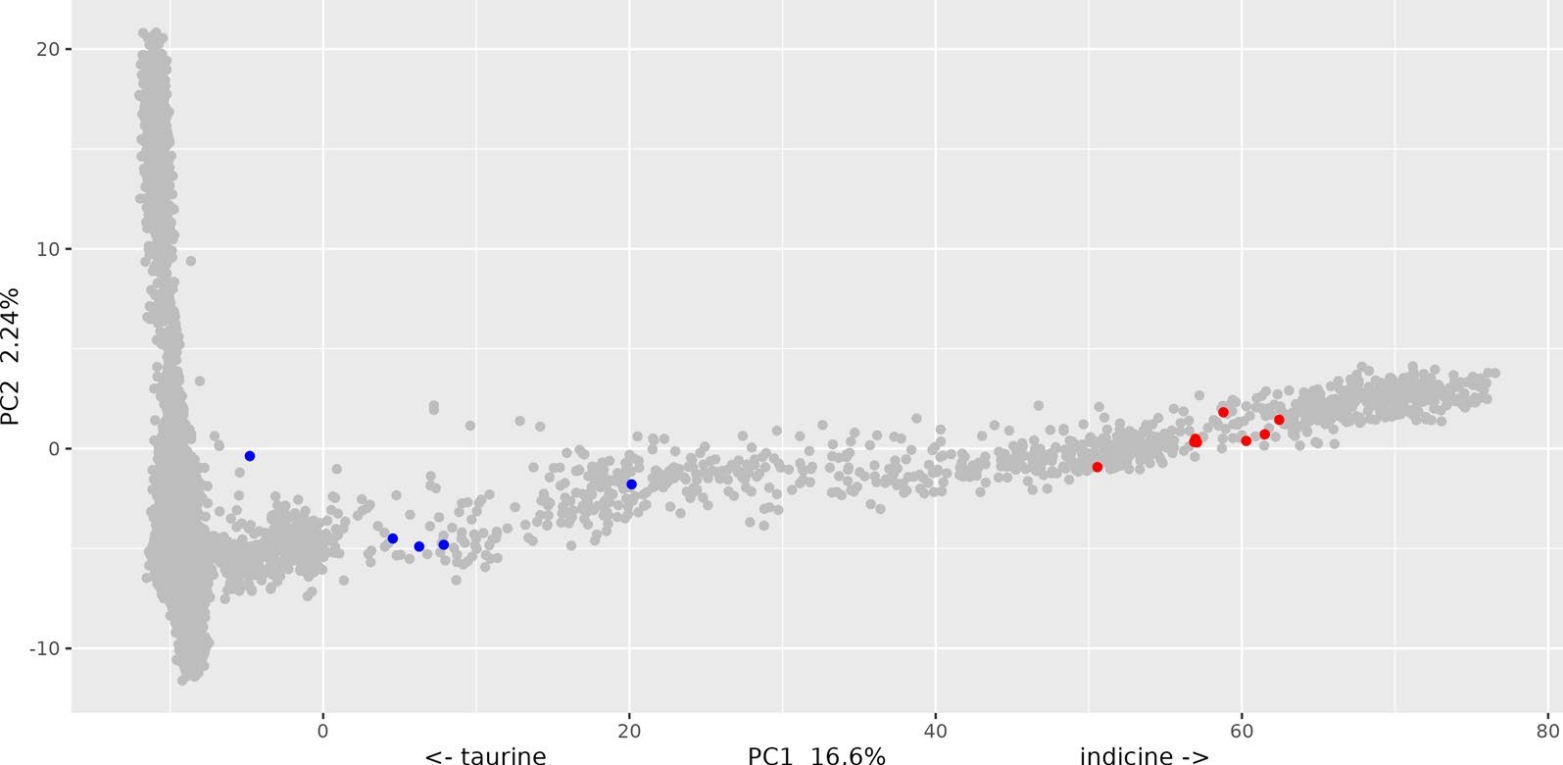
PCA based on 50k randomly selected genomic positions, displaying the breeds Bohai Black (blue) and Wenshan (red) remote from their submitter-assigned subspecies *B. indicus* (Bohai Black) and *B. taurus* (Wenshan), respectively

**Fig. 3:**
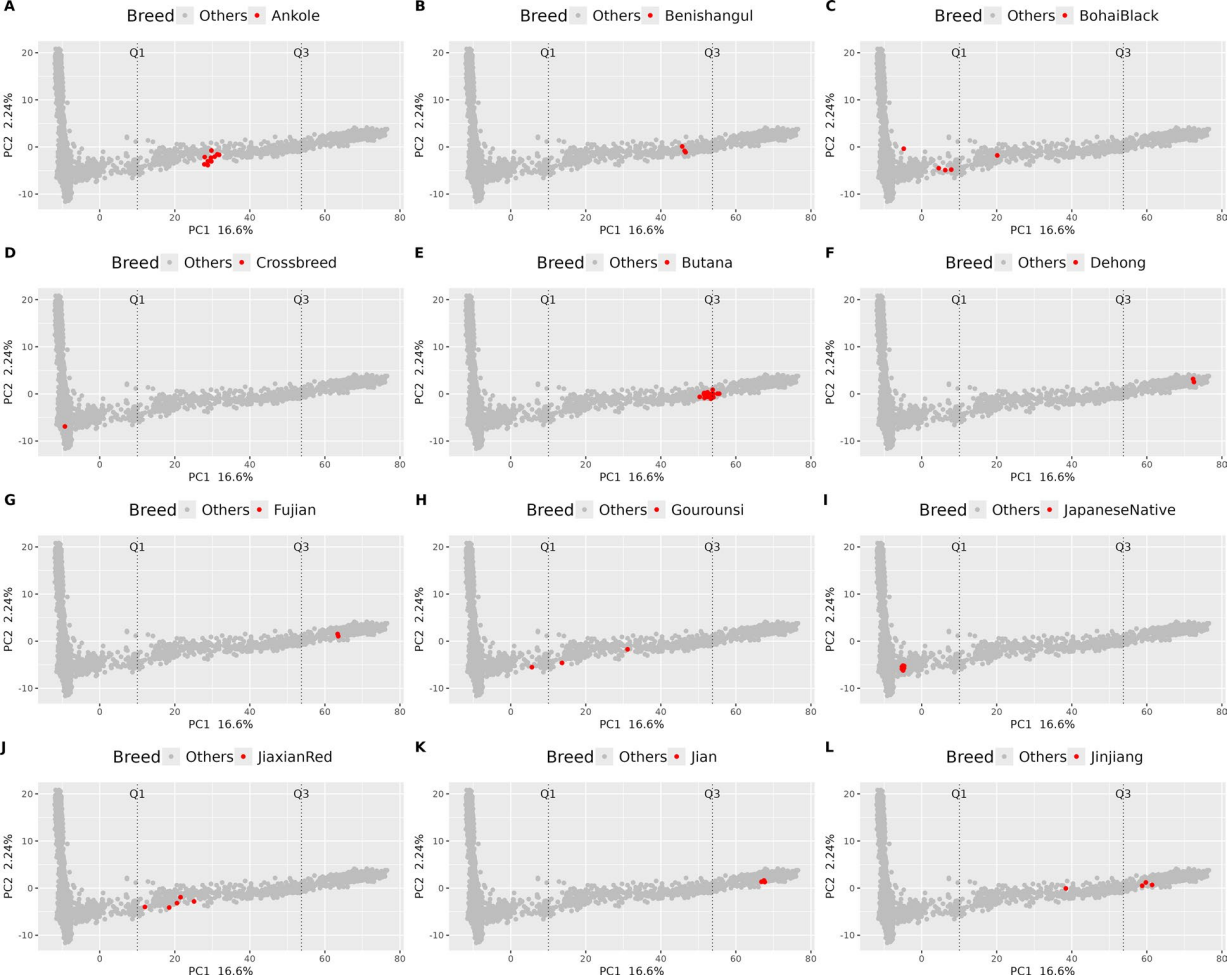
Visualization of the first two components of the PCA conducted on 50k randomly selected genomic positions, highlighting breeds with no clear subspecies assignments (**A** – **L**) with display of the quartile boundaries (Q1 and Q3) for the subspecies

**Fig. 4:**
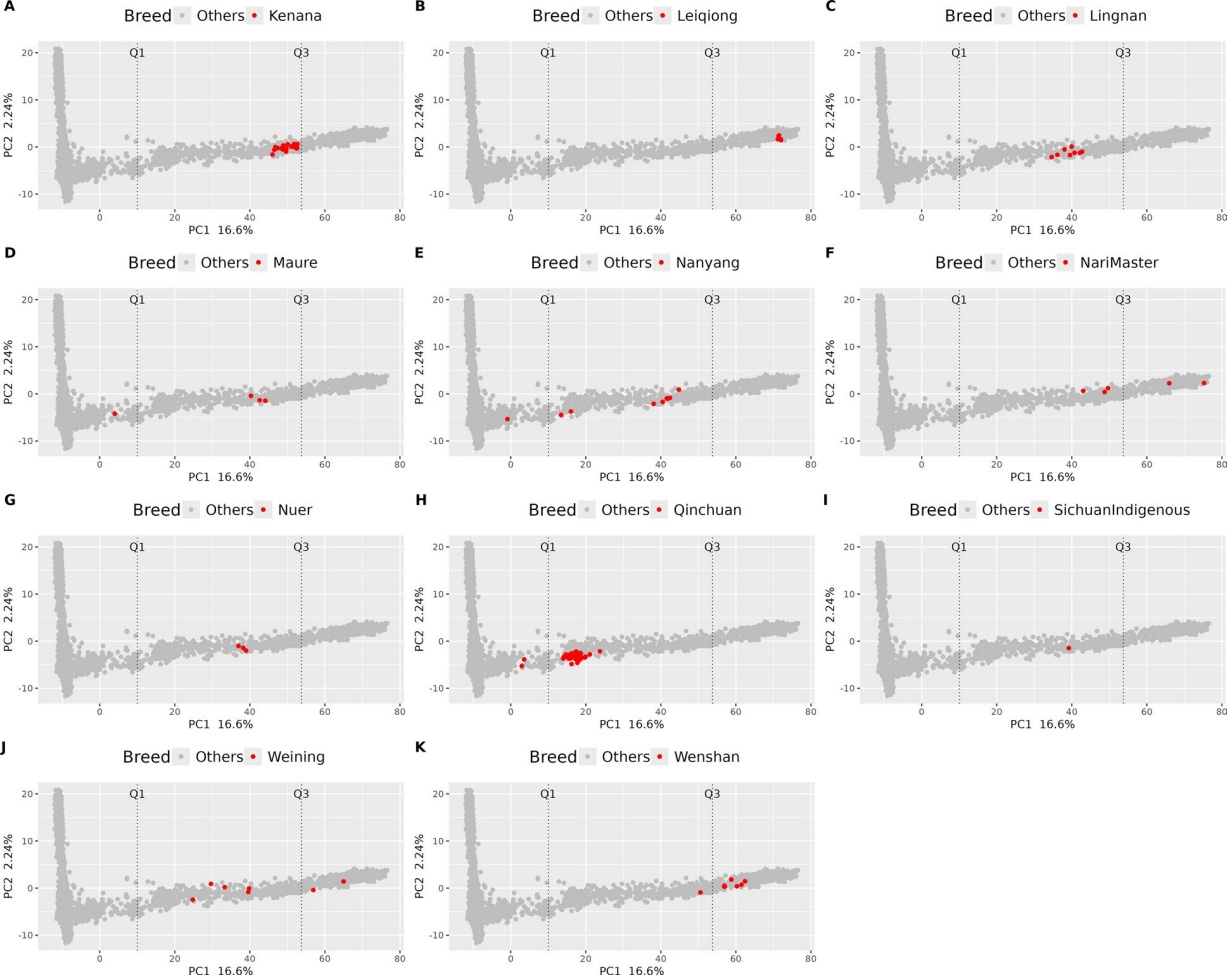
Visualization of the first two components of the PCA conducted on 50k randomly selected genomic positions, highlighting breeds with no clear subspecies assignments (**A** –**K**) with display of the quartile boundaries (Q1 and Q3) for the subspecies

**Table 3:** Comparison of the submitter-assigned subspecies, SCOPE results, and average ancestry per breed arranged by the average proportion of indicine admixture

No	Breed	Assigned subspecies	SCOPE result	Average indicine admixture	No. misassignments
1	JapaneseNative	Predicted Taurus/other	*B. taurus*	0.00	9
2	Crossbreed	other	*B. taurus*	0.00	1
3	BohaiBlack	*B. indicus*/*B. taurus*	*B. taurus*	0.17	3
4	Gourounsi	*B. taurus*	TaurusXIndicus	0.28	3
5	Qinchuan	*B. taurus*	TaurusXIndicus	0.29	39
6	Jiaxian Red	Other	TaurusXIndicus	0.31	5
7	Ankole	*B. indicus*	TaurusXIndicus	0.45	10
8	Nanyang	*B. taurus*/TaurusXIndicus	TaurusXIndicus	0.46	2
9	Maure	*B. indicus*	TaurusXIndicus	0.48	4
10	Lingnan	Other	TaurusXIndicus	0.55	8
11	Nuer	Other	TaurusXIndicus	0.56	3
12	Sichuan Indigenous	Other	TaurusXIndicus	0.57	1
13	Weining	*B. indicus*/TaurusXIndicus	TaurusXIndicus	0.59	5
14	Benishangul	Other	TaurusXIndicus	0.64	3
15	Kenana	*B. indicus*/*B. taurus*	*B. indicus*	0.71	4
16	Butana	*B. indicus*/*B. taurus*	*B. indicus*	0.73	2
17	Jinjiang	Other	*B. indicus*	0.74	4
18	Wenshan	*B. taurus*	*B. indicus*	0.78	8
19	Fujian	TaurusXIndicus	*B. indicus*	0.85	2
20	Jian	Other	*B. indicus*	0.89	4
21	Leiqiong	*B. taurus*/Other	*B. indicus*	0.94	5
22	Dehong	TaurusXIndicus	*B. indicus*	0.97	2
23	NariMaster	TaurusXIndicus	*B. indicus*	0.98	5

The thresholds for admixture proportions assign individuals with $\le$ 20 % indicine admixture to *Bos taurus*, with 20–70% indicine admixture to Taurus × Indicus, and those with $\ge$ 70% indicine admixture to *Bos indicus*. While the dataset under study covers a wide range of European taurine to indicine Asian breeds, the determined thresholds are to some degree specific to this dataset. In the metadata, the breeds Bohai Black and Wenshan are assigned to *B. indicus* and *B. taurus*, respectively. Our analysis shows that both breeds are separated from their assigned subspecies at the opposite ends of PC1 (see Fig. 2) and carry large proportions of the subspecies with which they cluster in the PCA (see Fig. 6 and Table 1). According to our framework this is a misassignment of the subspecies *B. taurus* and *B. Indicus*, which we consider to be a severe misassignment. The breed Ankole is labeled as *B. indicus* in the metadata, while the PCA displays the breed between the two subspecies and SCOPE shows almost equal proportions of admixture (see Figs. 3 and 6 and Table 3), which also reflects the breed’s assignment to the African Sanga group in literature [5, 36]. Assigning the breed to the subspecies TaurusXIndicus is a better reflection of its genomic information than the submitter assigned *B. indicus*. Misassigned subspecies were detected and corrected in 23 breeds (see Figs. 3, 4, and 6 and Table 3). In cases where more than one sub-species was assigned, the misassigned subspecies may be more frequent than the true subspecies, as in the case of Bohai Black and Leiqiong. A simple correction approach that selects the most frequently assigned subspecies from the metadata results in even more misassignments. This highlights the advantage of using genomic data in the misassignment detection framework.

**Fig. 5:**
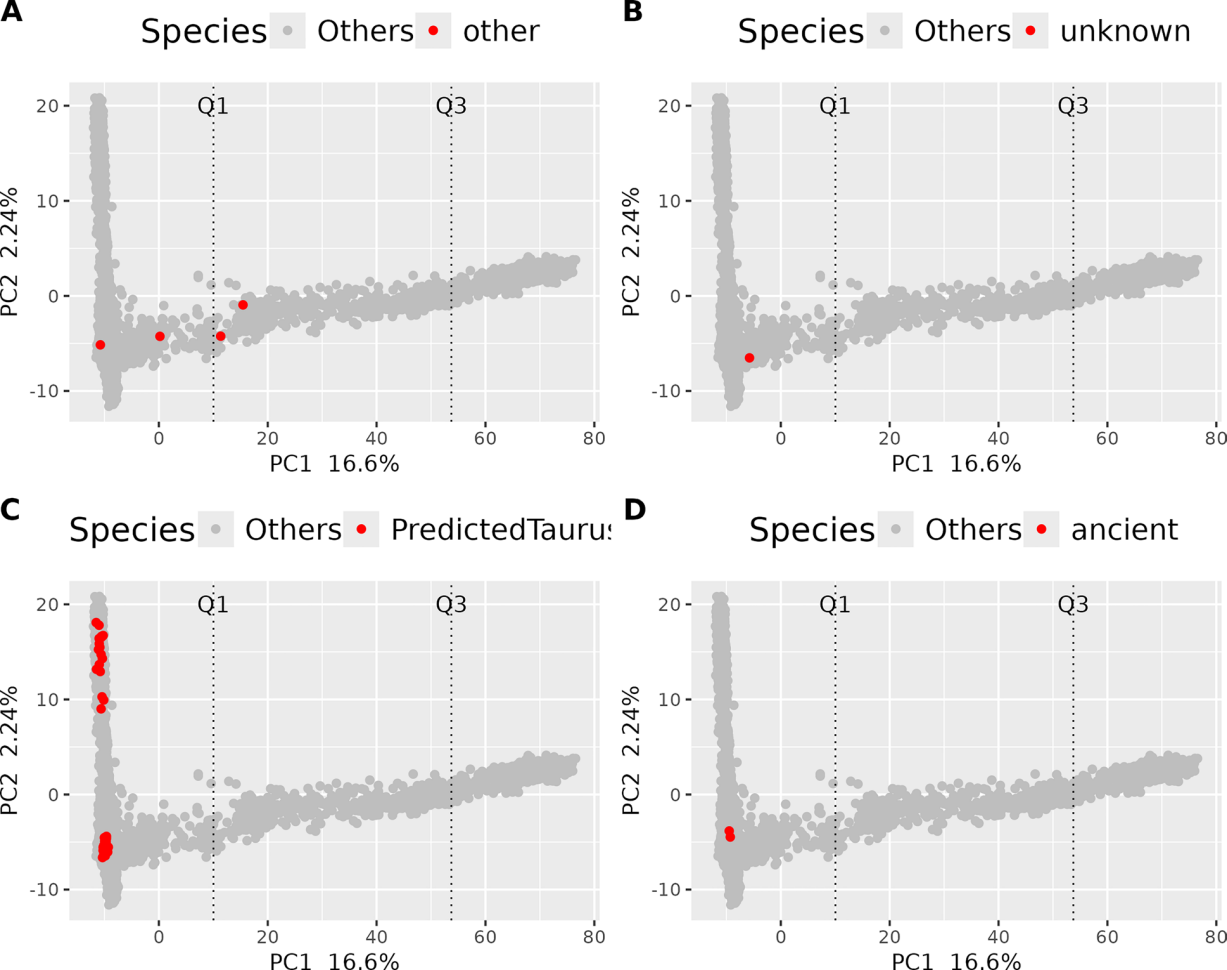
PCA for individuals with the subspecies "other, "unknown" and "PredictedTaurus", with display of the quartile boundaries (Q1 and Q3) for the subspecies

**Fig. 6:**
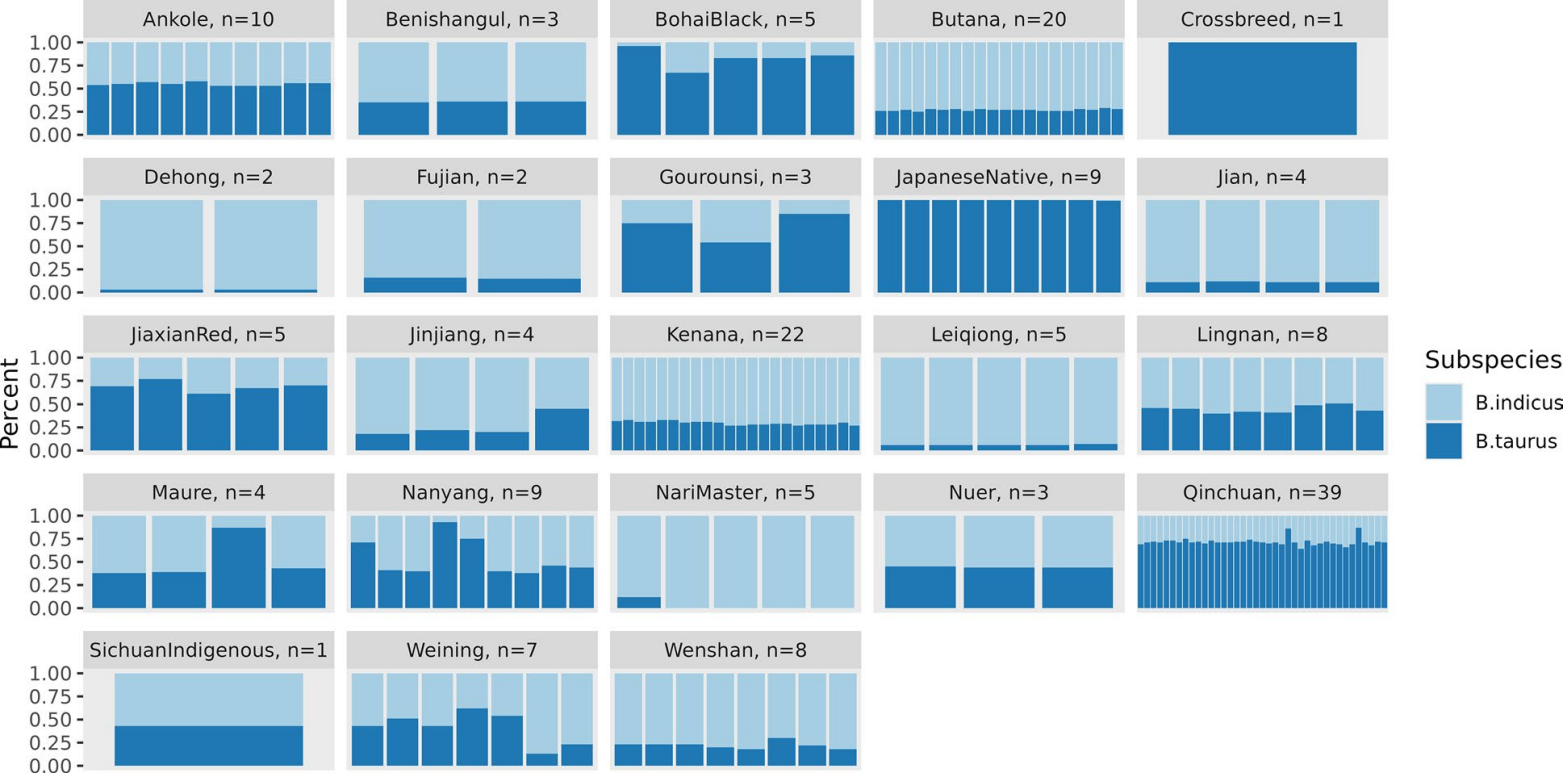
Analysis of ancestral populations for breeds with missing or presumably missassigned subspecies setting k=2 for *B. taurus* (dark-blue) and *B. indicus* (light-blue)

**Fig. 7:**
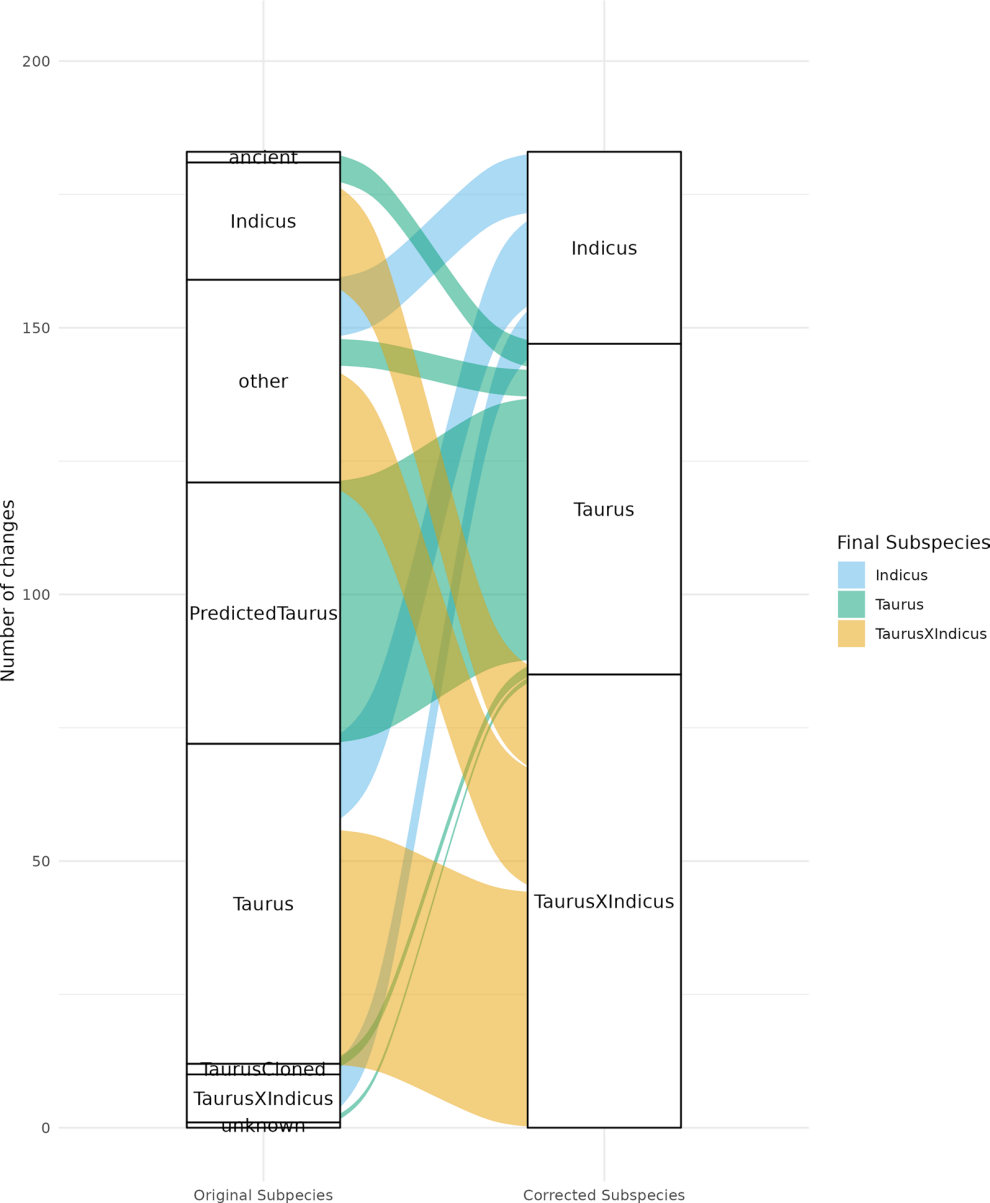
Flow chart of changes between the originally assigned subspecies and the subspecies assignment using the metadata analysis framework

As the subspecies thresholds reflect admixture proportions, we employed them to assign meaningful subspecies information to the small subspecies groups and individuals without breed information based on their PC1 positions. This confirmed the *B. taurus* subspecies for individuals assigned the term "PredictedTaurus" (n=42), "ancient" (n=2), and "unknown" (n=1) (see Fig. 5, Fig. 7). The remaining individuals assigned "other" in the original metadata are split between the *B. taurus* (n=2) and TaurusXIndicus (n=2) subspecies. Cloned *B. taurus* individuals are considered as *B. taurus*. After these corrections, the *B. taurus* remains the prevalent subspecies (n=5206), followed by *B. indicus* (n=620), TaurusXIndicus (n=364), and auroch (n=1).

### Breed-level misassignments

Several additional breeds were uncovered based on querying of the NCBI records. Some of these breed names either display high similarity to existing breeds or suggest the presence of additional breeds under currently existing groupings. We identified a total of 41 cases of potential misassignments between breeds. This includes both breeds that were part of the original metadata and breeds that were added to the metadata through the recovery process. To highlight the diversity of metadata errors, we distinguished between cases of breed splitting and merging, illustrating the range of misassignment types encountered.

**Table 4:** Table of cattle with discrepancies in the breeds stored in the metadata and the corresponding NCBI BioSamples

No	Metadata breed	BioSample breed	No. animals	Consequence	BioProject	References
1	JapaneseNative	Mishima	8	additional breed	PRJDB2660	[37]
2		Kuchinoshima	1	additional breed	PRJDA48395	[60]
3	IranAdmixed	Rashoki	9	additional breed	PRJEB5462	
4	Crossbreed	Maine Anjou/Beefbooster	7	additional individuals	PRJNA176557	[39]
5		Composite/Beefbooster	18	additional individuals	PRJNA176557	[39]
6		Blonde d’Aquitaine/Beefbooster	1	additional individual	PRJNA176557	[39]
7		Breed Crosses specified	5	additional breeds	PRJNA176557	[39]
8		Breed Crosses specified	1	additional breed	PRJNA256210	
9		Breed Crosses specified	1	additional individual	PRJNA256210	
10	UgandaAdmixed	Ankole	8	additional individuals	PRJEB1829	
11		AnkoleZebucross	2	additional breed	PRJEB1829	
12		EastAfricanShorthornZebu	4	additional breed	PRJEB1829	
13		ShorthornZebu	5	additional breed	PRJEB1829	
14		SmallEastAfricanZebu	2	additional breed	PRJEB1829	
15		Nganda	1	additional breed	PRJEB1829	
16		Nkiga	1	additional breed	PRJEB1829	
17		Nsongora	1	additional breed	PRJEB1829	
18		Ntoro	1	additional breed	PRJEB1829	

Concerning breed splits, we detected five erroneous groupings of breeds. The samples of the breed "Japanese Native" are labeled as Mishima and Kuchinoshima in the corresponding NCBI records (see Table 2). Previously, Tsuda et al. [37] considered Mishima and Kuchinoshima as separate breeds [37]. Furthermore, all but one individual of the breed "IranAdmixed" are stored in NCBI as "Rashoki", while the breed "UgandaAdmixed" comprises a variety of breeds according to the corresponding NCBI records (see Table 4). In addition, information on breeds or specific crossbreed proportions was retrieved for individuals that were recorded as Crossbreed in the metadata. All groups were split on the basis of this information.

Apart from the identification of additional breeds, the expansion of existing breeds breeds is likewise important, considering the Food and Agriculture Organization’s (FAO) recommendation to use $\ge$ 25 individuals [38] (page 14) when estimating allele frequencies of a breed. Previously, the Maine Anjou breed did not meet the requirement set for the sample size in the analysis of diversity metrics [12]. Therefore, the current misclassification in the metadata prevented further insight, although it should be noted that the additional seven individuals are not purebred [39].

**Table 5:** Cattle breeds with similar breed names, ordered by categories of potential misassignment sources

No.	Breed 1	Breed 2	No. breed 1	No. breed 2	Sum of animals	Decision
Inversion of Name Components						
1	Angus Red	Red Angus	33	20	53	Join
2	Ayrshire Finnish	Finnish Ayrshire	45	12	57	Join
Extensions						
3	Boran	Ethiopian Boran	21	10	31	No join
4	Danish Red Dairy	Modern Danish Red	4	54	58	Join
5	Danish Red Dairy	Traditional Danish Red	4	15	19	No join
6	Fjäll	Fjäll Cattle	11	6	17	Join
7	German Red Angler	Modern Angler	6	20	26	No join
8	German Red Angler	Traditional Angler	6	5	11	Join
9	Holstein	Holstein Friesian	1148	42	1190	Join
10	Kalmyk	Kalmykian	3	10	13	Join
11	Kazakh	Kazakh Whiteheaded	9	5	14	No join
12	Lithuanian Red	Traditional Lithuanian Red	12	4	16	No join
13	Modern Angler	Traditional Angler	20	5	25	No join
14	Modern Danish Red	Traditional Danish Red	54	15	69	No join
15	Swedish Red	Swedish Red Polled	50	6	56	No join
16	Tharparkar	Tharparker Modern	6	1	7	Join
17	Wagyu	Wagyu Modern	29	1	30	Join
Language issues						
18	Belgian Blue	Belgium Blue	9	1	10	Join
19	Tyrolean Grey	Grey Cattle	17	6	23	Join
20	Tyrolean Grey	Tyrolean Grauvieh	17	9	26	Join
21	Tyrolean Grauvieh	Grey Cattle	9	6	15	Join
Typographical errors						
22	Sahiwal	Shaiwal	6	1	7	Join
23	Muturu	Mututu	10	4	14	Join

**Fig. 8:**
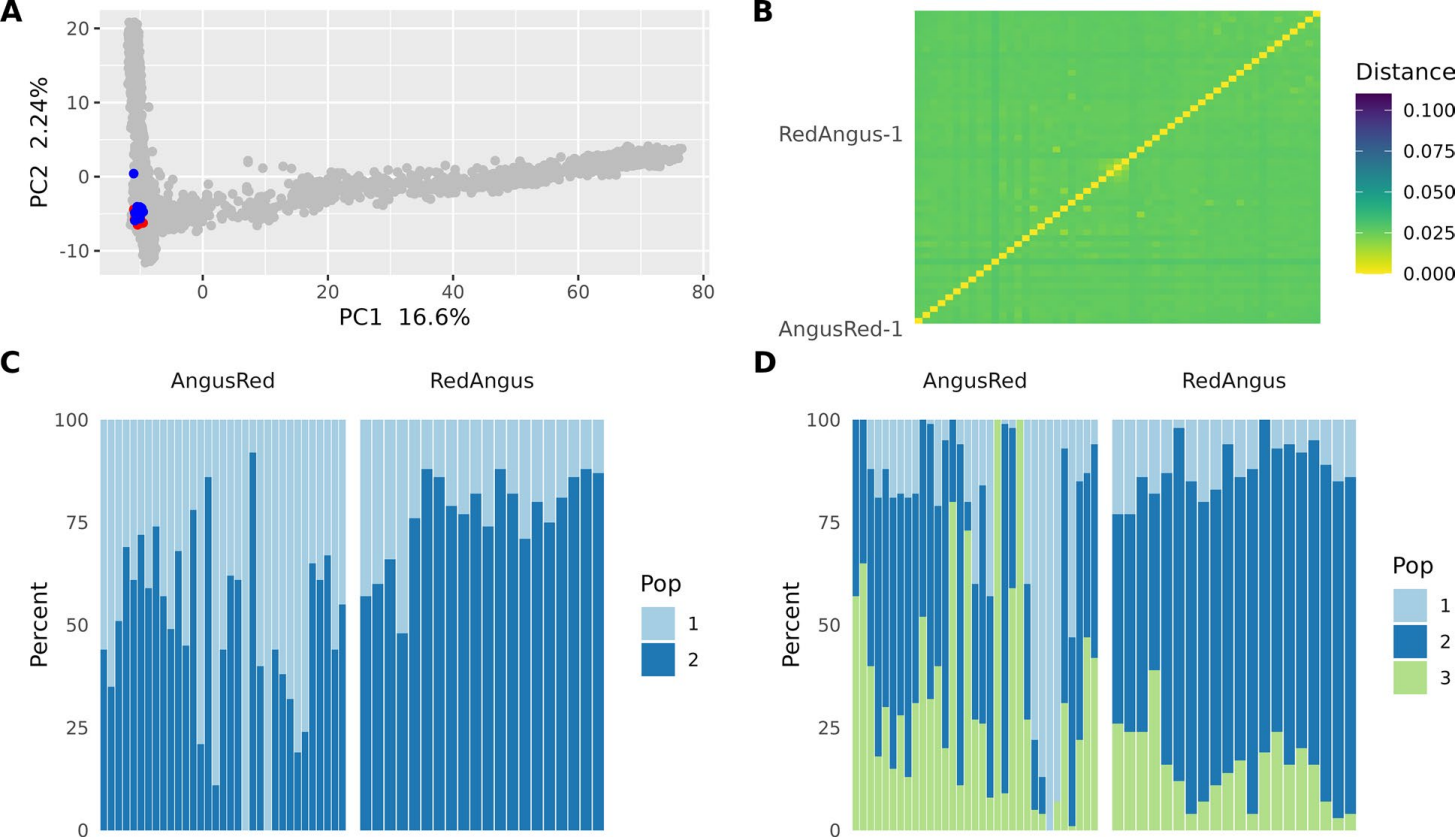
Analysis of the Red Angus and Angus Red populations by **A** the display of Red Angus (red) and Angus Red (blue) in the PCA of all individuals based on 50k genomic positions, **B** the genetic distance (1-IBS) between the individuals with labels for the first individuals of each breed, and the population admixture with *SCOPE* setting **C** k=2 and **D** k=3

**Fig. 9:**
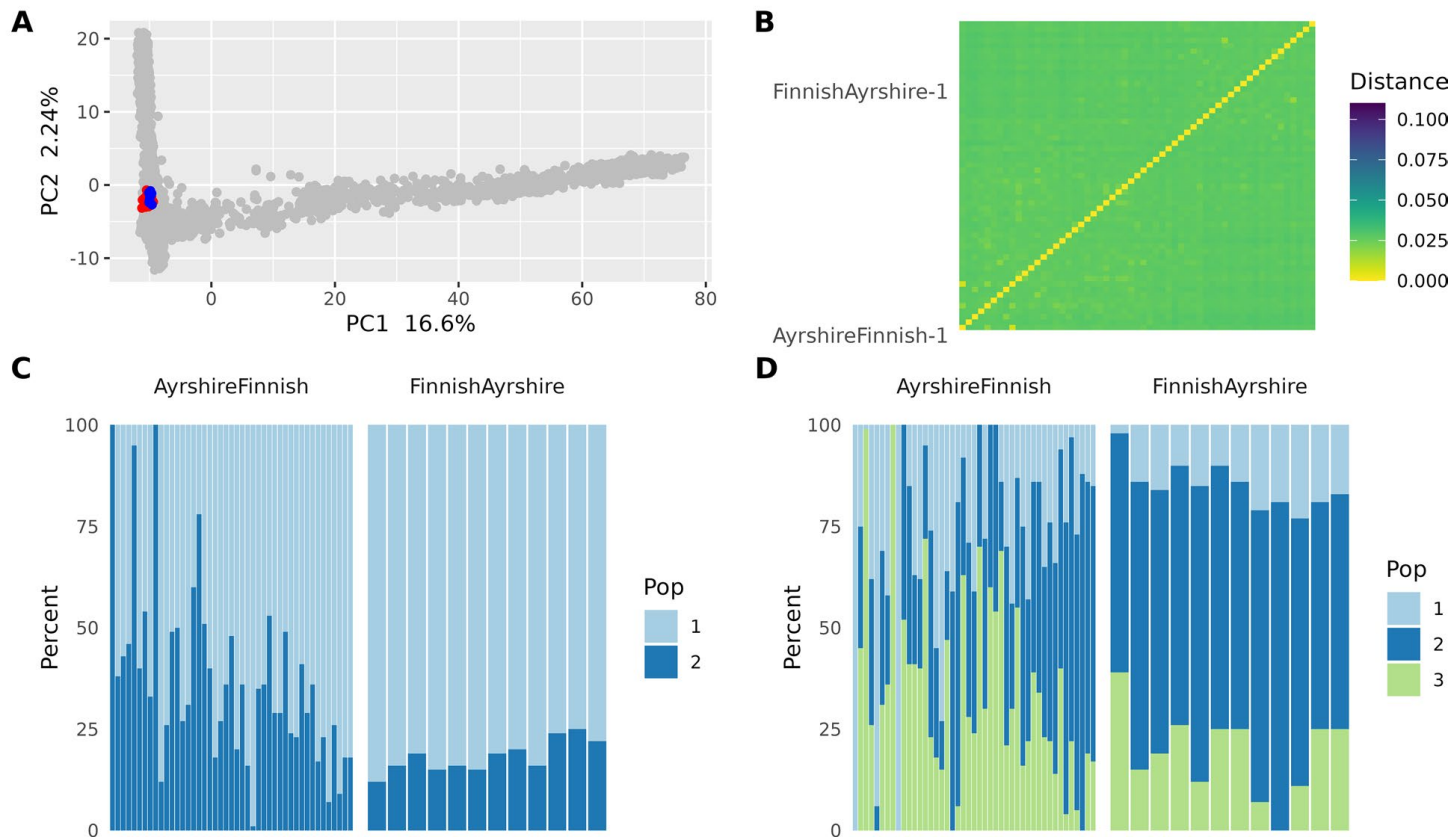
Analysis of the Finnish Ayrshire and Ayrshire Finnish populations by **A** the display of Ayrshire Finnish (red) and Finnish Ayrshire (blue) in the PCA of all individuals based on 50k genomic positions, **B** the genetic distance (1-IBS) between the individuals with labels for the first individuals of each breed, and the population admixture with *SCOPE* setting **C** k=2 and **D** k=3

Breeds suspected of being incorrectly split into two or more groups are more common than incorrectly grouped breeds; we identified 23 such pairs (see Table 5). The similarities in breed names arise from four main sources: component inversions, name extensions, language mixes, and typographical errors. Red Angus and Angus Red (55 individuals in total), as well as Finnish Ayrshire and Ayrshire Finnish (57 individuals in total) have inverted name components. In the PCA, the individuals of both pairs cluster together (see Fig. 8 A and 9 A, and Supplementary Figures S16 C and D) and the population admixtures are similar (see Figs. 8 C – D and 9 C – D ). The two pairs should be merged and termed as Red Angus and Finnish Ayrshire, respectively, to follow the general convention of breed names. The Red Angus breed, like the Maine Anjou breed, was previously found to be of insufficient sample size for a population analysis [12, 38], which has changed after merging.

**Fig. 10:**
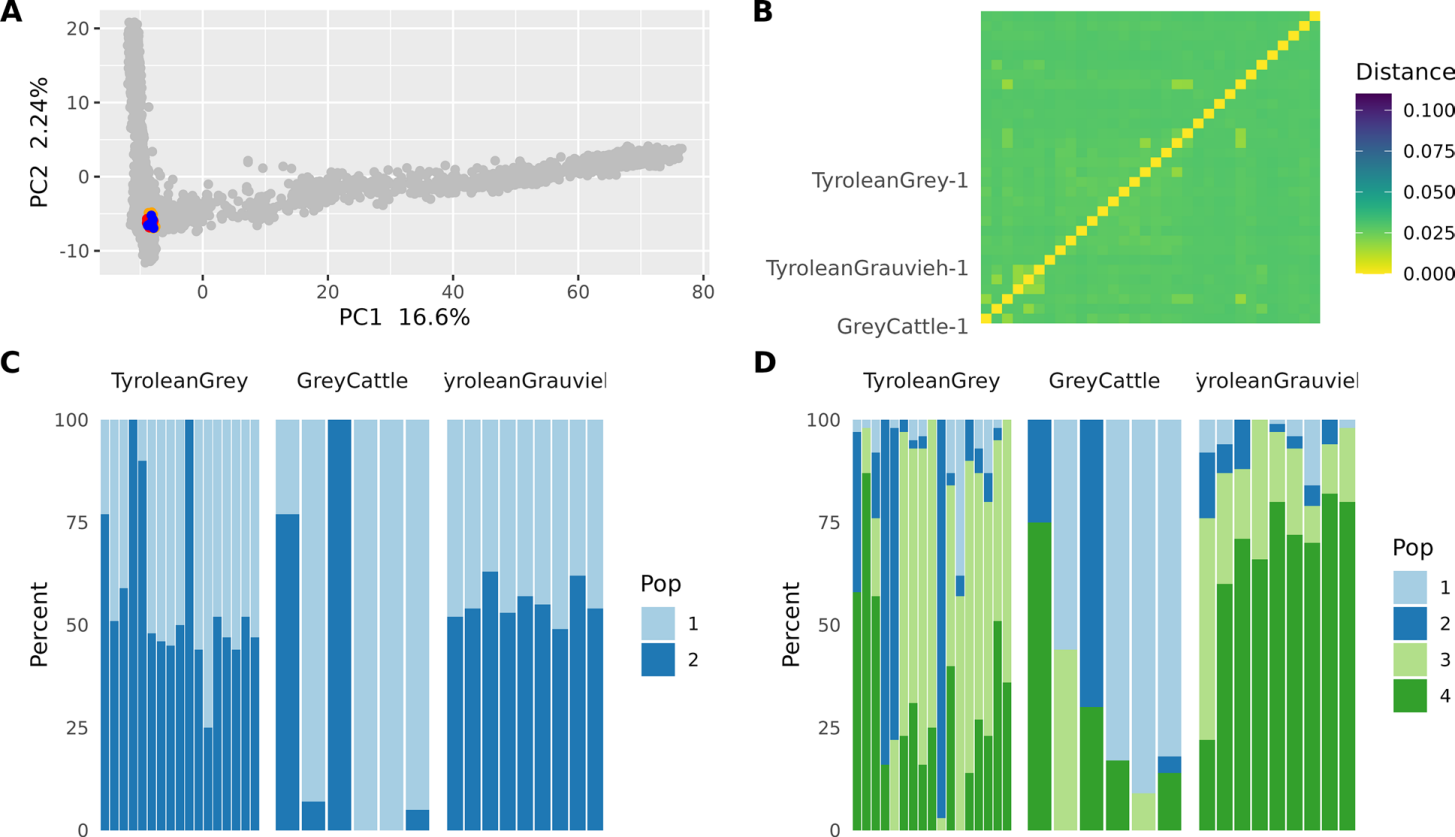
Analysis of the Tyrolean Grey, Tyrolean Grauvieh and Grey Cattle populations by **A** the display of Tyrolean Grey (orange), Tyrolean Grauvieh (blue) and Grey Cattle (red) in the PCA of all individuals based on 50k genomic positions, **B** the genetic distance (1-IBS) between the individuals with labels for the first individuals of each breed, and the population admixture with *SCOPE* setting **C** k=3 and **D** k=4

**Table 6:** Summary of misassignments assessed on different metadata levels. No subspecies information was recovered from NCBI, hence recovery data are not applicable at that level

	Subspecies level	Breed level	Sex
Recoveries from the NCBI	Not applicable	259	282
Misassignments	183	1635	2
Remaining unknown	0	101	551

We found two cases of typographical errors: Sahiwal – Shaiwal and Muturu – Mututu. The Sahiwal – Shaiwal individuals show some variation in PCA (see Supplementary Figure S1). The breed Shaiwal in the metadata is identical to the corresponding NCBI BioProject (PRJEB31621), while the same sample is referred to as Sahiwal in the corresponding publication [40], the original publication [41] and BioProject (PRJNA379859). In addition to correcting the apparent typing error in both the metadata and the NCBI, we suggest adding the original publication to the metadata to enhance transparency. Furthermore, the visible distances between the Sahiwal individuals in the PCA should be investigated in a dedicated study. The only breed pair, which resulted from the inclusion of information from the breed recovery step is the Mututu – Muturu pair, which partially overlaps in the PCA (see Supplementary Figure S2). Moreover, the Mututu samples are referred to as Muturu in the original publication linked to the NCBI data [42]. Both cases indicate that the presence of two breeds is based on a typographical error, which should be corrected, not only in the present dataset, but also in the corresponding NCBI records and the respective groups should be merged in the curated metadata.

#### Breed name extensions

Compared to name inversions, misassignments due to name extensions are more variable, as there is no uniform extension style (see Table 5). Moreover, these expand from pairs to triplets. In the group of name extensions two triplets are: German Red Angler—Traditional Angler—Modern Angler, and Traditional Danish Red—Modern Danish Red—Danish Red Dairy.

The breeds with the common term "Angler" overlap in the PCA, and show slight signs of structural genetic dissimilarity (see Supplementary Figures: S3 and S17 E). However, these do not necessarily apply to all individuals of the respective breeds. The Modern Angler breed shows heterogeneous proportions of admixture and while several individuals reflect the Traditional and German Red Angler, some individuals show diverging proportions (see Supplementary Figures S3 C–D, and S17 E). Given this heterogeneity, we decided to combine only the German Red Angler and Traditional Angler. In the triplet with the common term "Danish Red", the Traditional Danish Red display a higher genetic distance to the other two groups and different proportions in the ancestral populations (see Supplementary Figure S4 and S16 F). As a result, only the Modern Danish Red and Danish Red Dairy are merged and termed Modern Danish Red. Further, the pairs Fjäll— Fjäll cattle, Holstein—Holstein Friesian, and Wagyu—Wagyu Modern are identified and we propose the joining of each pair, given their high similarities in the PCA, and admixture proportions, along their low genetic distances (see Supplementary Figures S5, S6, S7, S17 F, and S18 C and D).

The pair Tharparkar – TharparkerModern combines two sources of error: an extension term error and a typing error. Again, both the 1000 Bull Genomes Project metadata and the information in the referenced NCBI record are incorrect (PRJEB31621), while the related publication [40], the original publication [41] and the original BioProject (PRJNA379859) denote the individual as Tharparkar. Similar to the Sahiwal individuals, the variability shown in the PCA (see Supplementary Figures S1 and S16 A) should be analysed in a dedicated study. Due to small sample sizes, the two Tharparkar breeds were pooled with two additional B. indicus breeds to meet the minimum sample size required for SCOPE analysis. Considering our analysis (see Supplementary Figures S1, S8, and S16 B), we propose to merge the two breeds under the correct breed Tharparkar.

#### Language issues

The breeds Belgian Blue and Belgium Blue show a linguistic error, as the two breeds exhibit high similarities in all conducted analyses (see Supplementary Figures S2, S7, and S17 B), and should be corrected to one breed under the name Belgian Blue. The pair Tyrolean Grey (NCBI: Tyrolean Grey or Tyrolean Grey Cattle [PRJEB11962, PRJEB18113]) – Tyrolean Grauvieh (NCBI: Tiroler Grauvieh [PRJEB28191]) displays a linguistic error due to a language mixture. Further, the breed Grey Cattle extends the pair to a triplet, based on the information from the corresponding NCBI records (NCBI: Tyrolean Grey Cattle [PRJEB28191]). The mix of German and English terms, on the one hand, and the description Grey Cattle, although the NCBI records are assigned the title Tyrolean Grey Cattle, on the other hand, make the identification of similar breed names difficult. All three breeds are displayed close in the PCA, but some variation in population admixture is visible especially between Grey Cattle and the other two (see Fig. 10 C–D). Given the information from the NCBI and the PCA alone, the samples should be merged. Considering the diverging results of the admixture analysis at k=2 and k=4, our framework points towards the question how the different sub-populations of this particular breed were bred. However, since diversity is a necessary prerequisite of livestock breeds, and the majority of indicators point to one breed, the present breeds should be merged under the name Tyrolean Grey.

Apart from similarities in breed names, the information from the NCBI BioSamples database indicates the presence of wrong breed assignments in 42 cases (see Supplementary Table S1), where the metadata assigns a different breed than the corresponding NCBI BioSample. Following our framework, we identify 19 individuals to be misassigned in the metadata (see Supplementary Figures S10, S11, S12, S13, S14, S15) and five presumable misassignments in the BioSamples database. Moreover, in one case both assignments are equally valid, as the ancestral populations of Swiss Fleckvieh align with a cross between Holstein and Simmental cattle (see Supplementary Figure S15). The misassignments identified here are particularly severe, as they go unnoticed without a targeted comparison between the metadata and the BioSamples database.

The reported sex differs in eleven samples when comparing the metadata to the BioSamples database (see Supplementary Table S2). In two cases, the Y chromosome supports the presence of a misassignment in the metadata (see Supplementary Figure S9). Consequently, the misassignments affect both the metadata and the information in the BioSamples entries equally. In addition, the query of the BioSamples database obtained additional information on the sex of 282 individuals.

Considering all analyses, 603 individuals carried misassignments on the breed or subspecies level, which aligns with previously reported estimates of metadata error rates in RNA-sequencing data [15]. This number is lower than the number of total misassignments on the breed and subspecies levels (see Table 6), since some individuals were subjected to corrections on more than one level of the metadata. Additionally,in cases of the wrongful recognition of two breeds instead of one, all individuals were counted to be affected.

## Discussion

Community collection efforts, as the 1000 Bull Genomes Project, can on the one hand reduce the workload and financial burden of the individual contributors, on the other hand, they can be prone to irregularities and misassignments in the metadata. As a consequence of the potentially heterogeneous metadata, the need for curation efforts increases. Since metadata errors can never be ruled out and the growth in data availability fosters metadata errors [15], the provision of an interpretable and efficient pipeline to ensure data integrity rises, especially when working with incomplete metadata.

Our analysis highlights the importance of thorough metadata quality checks. Even in widely used datasets, like the 1000 Bull Genomes Project, metadata errors occur on different levels of the metadata and may affect downstream research applications.

The recovery of missing metadata through NCBI databases improved the metadata completeness, but heterogeneous recording practices hindered an easy access to the information and the establishment of a "one size fits all" solution to retrieve metadata on an individual’s breed from the NCBI. As a result, the findability and reusability of the information which is linked to the quality of the meta information [15, 43] are reduced. Likewise, reproducibility is reduced as it is related to the completeness of the metadata [43]. In our case study, this relates to the fact that only findable breeds can be analysed meaningfully by users of the dataset. Even difficult recoveries, as reported for the Qinchuan cattle, provide great value as it increases the sample size from two to 39 individuals, qualifying the breed for more in-depth population analyses.

The recovery of the breed information further impacts the detection of misassignments on the subspecies level. Several inconsistencies in the subspecies assignments become apparent only after the recovery of the breed. Previously, no meaningful grouping of the individuals was possible due to the uniform assignment "Unknown". While the importance of complete metadata has been discussed in the context of the FAIR practices [43], our analysis highlights the connection to metadata curation and the systematic detection of metadata misassignments.

### Subspecies

While taurine and indicine breeds can in general be distinguished phenotypically, there is no sharp boundary between them in a genomic level. Our results reflect previous observations linking PC1 to the subspecies under consideration [20, 30–33] with hybrids observed between the two subspecies [30]. We did not observe a clear differentiation between populations of the same subspecies by continent, unlike prior analyses (e.g. [20, 30, 32]).

Previously, Gao et al. [31] observed a linkage between the ancestry (at k=2) and first PC of their molecular analysis and the geographical north–south gradient in Chinese cattle. This analysis motivated us to perform a similar approach, linking the results of the PCA and the ancestral populations for a qualified assessment of misassignments. In contrast to Gao et al. [31], we are analysing several thousand individuals. Furthermore, we are interested in the general continuity of taurine and indicine ancestry on a global scale. Therefore, we sampled individuals evenly across PC1, generating samples of slightly smaller size compared to the analysis of Gao et al. [31]. This approach allows us to maintain the diversity we aim to present while reducing the computational burden and balance the uneven number of samples, as the subspecies are unbalanced within the dataset, with a majority of the individuals being taurine (see Table 1). Since we aim for an efficient analysis pipeline, it is important to note that this step is by far the most computationally intensive one in the entire framework.

The impact of misassignments at the subspecies level on downstream analyses depends on the specific use of the data. The taurine reference panel used for imputation is not affected, as it does not contain the specific individuals that we identify as misclassified. However, the use of the dataset based on the metadata can lead to both incorrect inclusion as well as incorrect exclusion of individuals when filtering for subspecies. With regard to cattle breeds, the exclusion of individuals results in smaller sample sizes. Our study shows that using genomic data to correct metadata misassignments helps to ensure that datasets can be more effectively reused by the scientific community.

### Breeds

The detection of misassignments on the breed level proved to be more challenging than the detection of misassignments on the subspecies level, as the heterogeneity of detected errors illustrates (see Table 5). Here, a two-fold hurdle had to be managed: (1) both the merging and splitting of breeds/groups as well as (2) the variable breed definitions/namings and the existing genetic variability within breeds. The latter two are known challenges for the recoding and error detection of metadata [15, 17].

Complex cases, as the Tyrolean Grey, Angler, and Danish Red, reflect the difficulty of merging animals on a breed level. While the individuals have highly similar breed names, the analysis of the genetic data displays a certain degree of diversity and dissimilarity between the individuals. Since introgressions associated with changes in admixture proportions are known in other red dairy breeds [44], a similar effect could be present in Modern Angler. However, as our framework is not designed to answer this question, further investigation using additional techniques, such as an analysis of molecular variance [45], should be undertaken in a dedicated study. It would be valuable to investigate the specific reason for the differences in population admixture in a more in-depth analysis.

As most livestock, cattle of different breeds are often crossed, e.g. the Piedmontese Normande is the result of a cross between a Piedmontese and a Normande individual (PRJEB18113). Consequently, there is no true extension to the breed name and the breeds remain separate in the metadata. In order to designate such cases, we recommend to add an "x" between the breeds, when combining them in one breed name, similar to the entry in the NCBI (PRJEB18113), the recommendations of Harrison et al. [17], and previous metadata of the 1000 Bull Genomes Project (see Supplementary Files 1 of [9]). This would make it possible to distinguish between pure- and crossbred animals at one glance, and make the metadata more accessible by improving their findability. Furthermore, not all similarities in the breed names lead to investigations beyond step four of our framework. The pairs Boran – EthiopianBoran, Kazakh – KazakhWhiteheaded, and SwedishRed – SwedishRedPolled form distinct clusters in the PCA, illustrating that similar breed names do not necessarily result from misassignment or do not reflect genetic similarity. Consequently, the breeds remain separate in the metadata.

The majority of the detected misassignments could be avoided by the employment of either a custom controlled vocabulary, or the implementation of the already existing livestock breed ontologies (LBO) from the FAANG database [17] in the metadata of future runs of the 1000 Bull Genomes Project. While the former could be implemented faster by a list of permitted breeds and subspecies, the latter could have a long-lasting positive impact, since a considerable proportion of the samples is already provided in the NCBI databases. In either case: the benefits of improved metadata quality call for the implementation of active searches for existing metadata misassignments and improved curation practices. Given the universality of metadata challenges, our framework offers a scalable solution applicable to other livestock and non-livestock genomic datasets.

As this study represents a case-specific application, the direct applicability of the presented framework to other datasets is limited. Not all datasets will allow for database queries to cross-validate metadata entries, and different metadata classes (e.g., population-related or technical metadata) will require distinct evaluation strategies beyond those demonstrated here. However, the outline of our framework can serve as a guide, since PCA and distance calculations (paired with hierarchical clustering) are techniques that are applicable to a wide variety of variables.

## Conclusions

This study emphasizes that clean, consistent metadata is essential for meaningful reuse of genomic datasets, even when obtained from established consortia. Therefore, its careful examination should not be foregone, even when working with well-established, prestigious datasets. We showcase how exploring big datasets in a data-driven manner can uncover hidden misassignments at different levels in the metadata. Given the rich genomic information, all missing subspecies assignments could be resolved. In total, misassignments on the breed or subspecies level were corrected for 603 individuals. In addition, our investigation provides further insight on the continuous gradient between the taurine and indicine subspecies, emphasizing the difficulty to establish hard boundaries between subspecies based on genomic information.

## Additional file

Supplementary Material 1.

Supplementary Material 2.

Supplementary Material 3.

Supplementary Material 4.

## Data Availability

The full run 9 dataset of the 1000 Bull Genomes Project is not publicly available as it is only available to 1000 Bull Genomes Consortium members. Metadata for publicly available samples in the project is included in Supplementary Table S3. The anonymized metadata, PCA, admixture analysis, and distance matrix can be made available upon reasonable request. The scripts used for the analysis shown in this manuscript are deposited under: https://github.com/azifiDils/Metadata_correction
