# Supplementary material for: Genomic solutions to metadata challenges: a case study on the 1000 Bull Genomes Project
Source: Genet Sel Evol. 2026 Jul 24;58:36. doi: 10.1186/s12711-026-01061-w (PMC13401786; doi:10.1186/s12711-026-01061-w)
Supplement: Supplementary file 1 — Supplementary Material 1. [file 12711_2026_1061_MOESM1_ESM.pdf]

Additional Files

Additional file 1: Figure S1
PCA and Distances matrices (1-IBS) of the breed pairs (A,B)Sahiwal –Shaiwal, (CD) Tharparkar – TharparkerModern.

Additional file 2: Figure S2
PCA and Distances matrices (1-IBS) of the breed pairs (A,B) Fjäll – Fjäll Cattle, (C,D) Muturu – Mututu , (E,F) Kalmyk – Kalmykian, (G,H) Belgian Blue – Belgium Blue, and (I,J) LithuanianRed – TraditionalLithuanianRed.

Additional file 3: Figure S3
Analysis of the Angler populations by (A) the display of Modern Angler (red), German red Angler (blue), and Traditonal Angler (orange) in the PCA of all individuals based on 50k genomic positions, (B) the genetic distance (1-IBS) between the individuals and the population admixture with *SCOPE* setting (C) k=2 and (D) k=4.

Additional file 4: Figure S4
Analysis of the Danish Red populations by (A) the display of Modern Danish Red (red), Traditional Danish Red (blue), and Danish Red Dairy (orange) in the PCA of all individuals based on 50k genomic positions, (B) the genetic distance (1-IBS) between the individuals and the population admixture with *SCOPE* setting (C) k=2 and (D) k=4.

Additional file 5: Figure S5
Analysis of the Holstein Friesian and Holstein (84 individuals sampled) populations by (A) the display of Holstein (red) and Holstein Friesian (blue) in the PCA of all individuals based on 50k genomic positions, (B) the genetic distance (1-IBS) between the individuals and the population admixture with *SCOPE* setting (C) k=2 and (D) k=3.

Additional file 6: Figure S6
Analysis of the Wagyu and Wagyu Modern populations by (A) the display of Wagyu Modern (red) and Wagyu (blue) in the PCA of all individuals based on 50k genomic positions, (B) the genetic distance (1-IBS) between the individuals and the population admixture with *SCOPE* setting (C) k=2 and (D) k=3.

Additional file 7: Figure S7
Combined admixture analysis of small taurine breeds, setting k=5 (A), and k=10 (B).

Additional file 8: Figure S8
Combined admixture analysis of small indicine breeds, setting k=4 (A), and k=9 (B).

Additional file 9: Figure S9
Assessment of sex, based on missing positions on the Y-chromosome.

Additional file 10: Figure S10
Analysis of the individuals named Crossbreed in the metadata and Composite in the BioSamples entries by (A) the display of Crossbreed (red) and Composite (blue) populations in the PCA of all individuals based on 50k genomic positions, (B) the genetic distance (1-IBS) between the individuals and the population admixture with *SCOPE* setting (C) k=2 and (D) k=3, ordering the individuals by the admixture proportions shown at k=2.

Additional file 11: Figure S11
Analysis of the individuals named Gelbvieh in the metadata and Beefmaster in the BioSamples entries by (A) the display of Gelbvieh (red) and Beefmaster (blue) populations in the PCA of all individuals based on 50k genomic positions, (B) the genetic distance (1-IBS) between the individuals and the population admixture with *SCOPE* setting (C) k=2 and (D) k=3, ordering the individuals by the admixture proportions shown at k=2.

Additional file 12: Figure S12
Analysis of the individuals named Brown Swiss in the metadata and Fleckvieh in the BioSamples entries by (A) the display of Fleckvieh (red) and Brown Swiss (blue) populations in the PCA of all individuals based on 50k genomic positions, (B) the genetic distance (1-IBS) between the individuals and the population admixture with *SCOPE* setting (C) k=2 and (D) k=3, ordering the individuals by the admixture proportions shown at k=2.

Additional file 13: Figure S13
Analysis of the individuals named Galloway in the metadata and Brown Swiss in the BioSamples entries, and individuals named Belted Cattle in the metadata and Galloway in the BioSamples entries by (A) the display of Galloway Belted (red) and Belted Cattle (blue) and Galloway (orange) populations in the PCA of all individuals based on 50k genomic positions, (B) the genetic distance (1-IBS) between the individuals. (C) Galloway (red) and Brown Swiss (blue) populations in the PCA of all individuals based on 50k genomic positions, (D) the genetic distance (1-IBS) between the individuals. Population admixture was conducted with *SCOPE* setting (E) k=2 and (F) k=4 and ordering the individuals by the admixture proportions shown at k=2.

Additional file 14: Figure S14
Analysis of the individuals named Charolais in the metadata and Simmental in the BioSamples entries by (A) the display of Charolais (red) and Simmental (blue) populations in the PCA of all individuals based on 50k genomic positions, (B) the genetic distance (1-IBS) between the individuals and the population admixture with *SCOPE* setting (C) k=2 and (D) k=3, ordering the individuals by the admixture proportions shown at k=2.

Additional file 15: Figure S15
Analysis of the individuals named Simmental in the metadata and Holstein in the BioSamples entries, individuals named Holstein in the metadata and Simmental in the BioSamples entries, and individuals named SwissFleckvieh in the metadata and Holstein or SimmentalxHolstein in the BioSamples entries by (A) the display of Holstein (orange), Simmental (blue), and Swiss Fleckvieh (red) populations in the PCA of all individuals based on 50k genomic positions, (B) the genetic distance (1-IBS) between the individuals and the population admixture with *SCOPE* setting (C) k=2 and (D) k=4, ordering the individuals by the admixture proportions shown at k=2.

Additional file 16: Table S1
Misassignments identified by comparing the metadata-reported breed with the breed information recorded in the corresponding NCBI BioSamples entries.

Additional file 17: Table S2
Overview of samples with inconsistencies between metadata sex and BioSample sex annotations.

Additional file 18: Figure S16
Phylogenetic trees based on genetic distances of individuals of breeds with highly similar names, including one further breed (Ogaden for indicine breeds and Shorthorn for taurine breeds) and one Auroch individual as reference (A) Sahiwal and Shaiwal (B) Tharparkar and Tharparker Modern, (C) Red Angus, Angus Red, (D) Finnish Ayrshire and Ayrshire Finnish, (E) Grey Cattle, Tyrolean Grauvieh, and Tyrolean Grey, (F) Danish Red Dairy, Modern Danish Red and Traditional Danish Red

Additional file 19: Figure S17
Phylogenetic trees based on genetic distances of individuals of breeds with highly similar names, including one further breed (Ogaden for indicine breeds and Shorthorn for taurine breeds) and one Auroch individual as reference (A) Muturu and Mututu (B) Belgian Blue and Belgium Blue, (C) Kazakh and Kazakh Whiteheaded (D) Lithuanian Red and Traditional Lithuanian Red, (E) German Red Angler, Modern Angler, and Traditional Angler, (F) Wagyu and Wagyu Modern.

Additional file 20: Figure S18
Phylogenetic trees based on genetic distances of individuals of breeds with highly similar names, including one further breed (Ogaden for indicine breeds and Shorthorn for taurine breeds) and one Auroch individual as reference (A) Swedish Red and Swedish Red Polled (B) Kalmyk and Kalmykian, (C) Fjäll and Fjäll Cattle (D) Holstein and Holstein Friesian.

Additional file 21: Figure S19
In all graphics, the subspecies B. taurus (red) and B.indicus (blue) are highlighted, while all remaining subspecies groups are displayed in grey. (A-D) Four independent repetitions of the principal component analysis of all individuals conducted on 50,000 randomly sampled variants.(E) Principal component analysis for all individuals conducted on 120k randomly smple variants. (F) Principal component analysis for all individuals conducted on 500k randomly sampled variants.

Additional file 22: Figure S20
(A-E) Density plot of individuals sampled evenly across PC1 in the analysis of ancestral populations (left) and density of admixture proportions of the evenly sampled individuals across PC1 in the analysis of ancestral populations (right) for all five repetitions of the analysis with SCOPE.

Additional file 23: Figure S21
In all graphics, the subspecies B. taurus (red) and B.indicus (blue) are highlighted, while all remaining subspecies groups are displayed in grey. The quartiles used as thresholds for the determination of subspecies, obtained from the analysis of ancestral populations, are displayed in black. (A) Principal component analysis of all individuals conducted on 50k randomly sampled variants of chromosome 29 with filtering for a minimum coverage of 10 reads prior to imputation, (B) Principal component analysis of all individuals conducted on 50k randomly sampled variants of chromosome 29 without any additional filtering steps, and (C) Principal component analysis of all individuals conducted on 50k variants randomly sampled across all chromosomes.

Additional file 24: Table S3
Metadata for publicly available samples

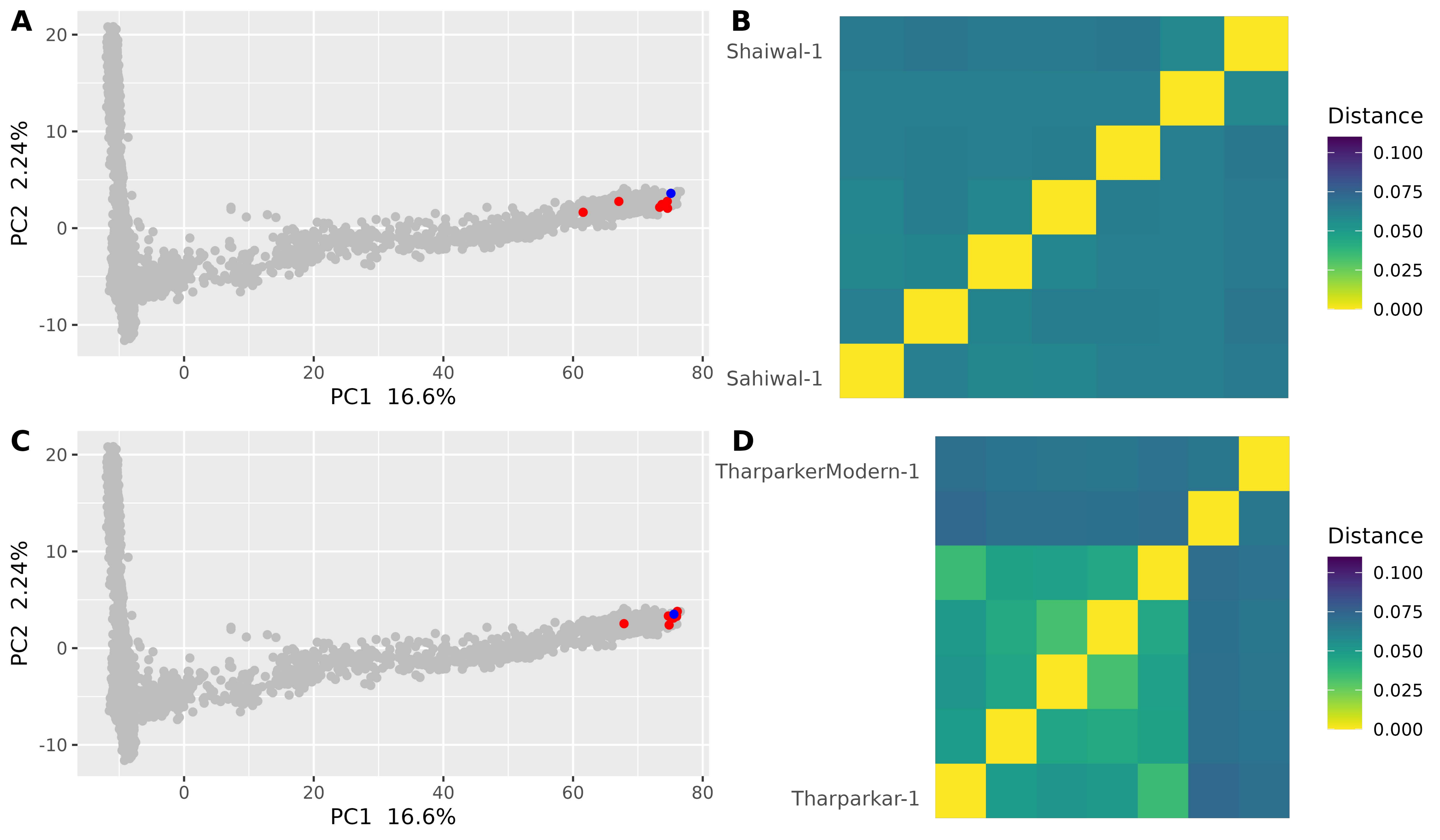

A
B
C
D
PC2 2.24%
PC1 16.6%
Shaiwal-1
Sahiwal-1
TharparkerModern-1
Tharparkar-1
Distance
0.100
0.075
0.050
0.025
0.000

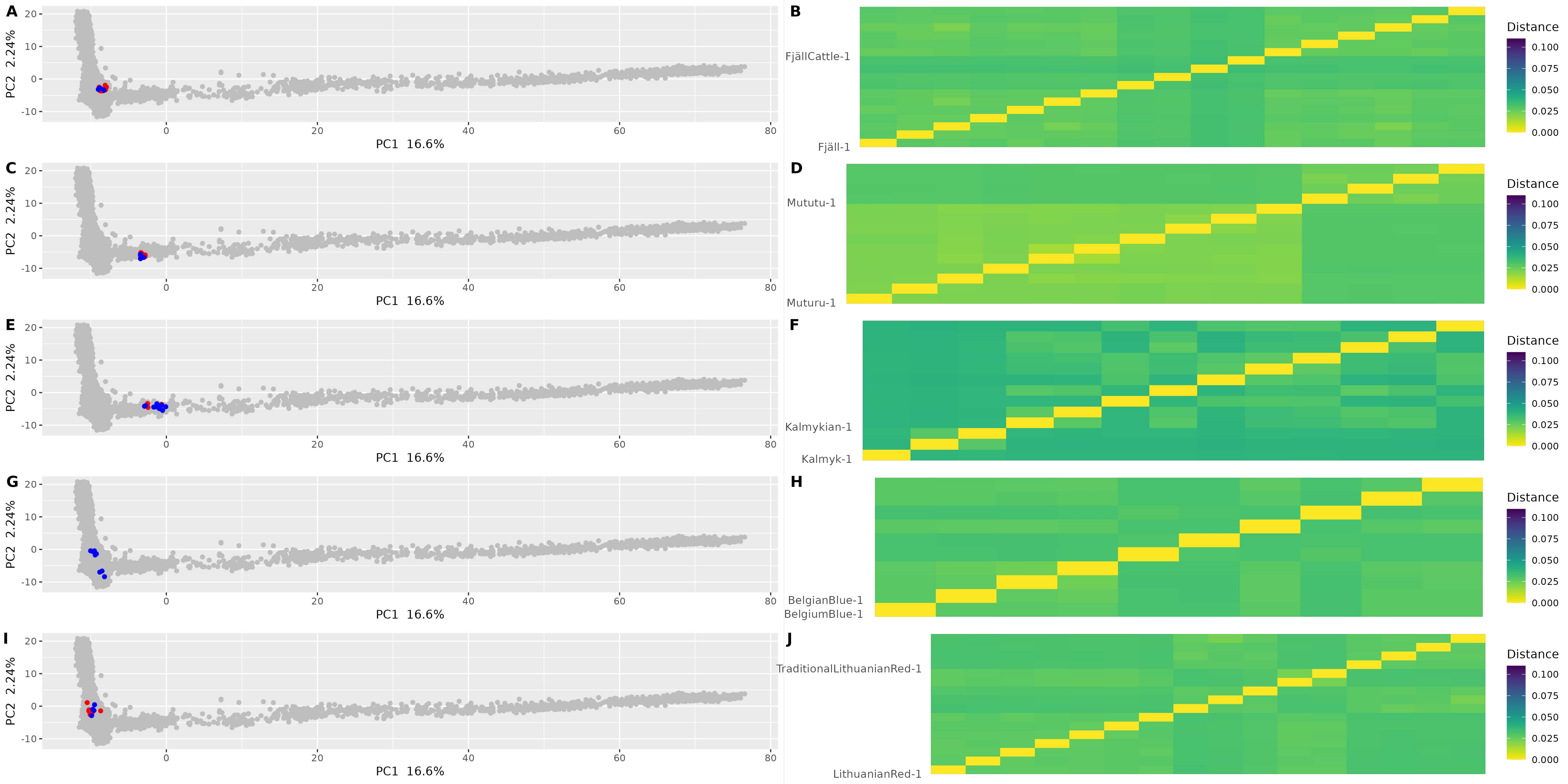

A
B
C
D
E
F
G
H
I
J
PC1 16.6%
PC2 2.24%
Distance
0.100
0.075
0.050
0.025
0.000
FjällCattle-1
Fjäll-1
Mututu-1
Muturu-1
Kalmykian-1
Kalmyk-1
BelgianBlue-1
BelgiumBlue-1
TraditionalLithuanianRed-1
LithuanianRed-1

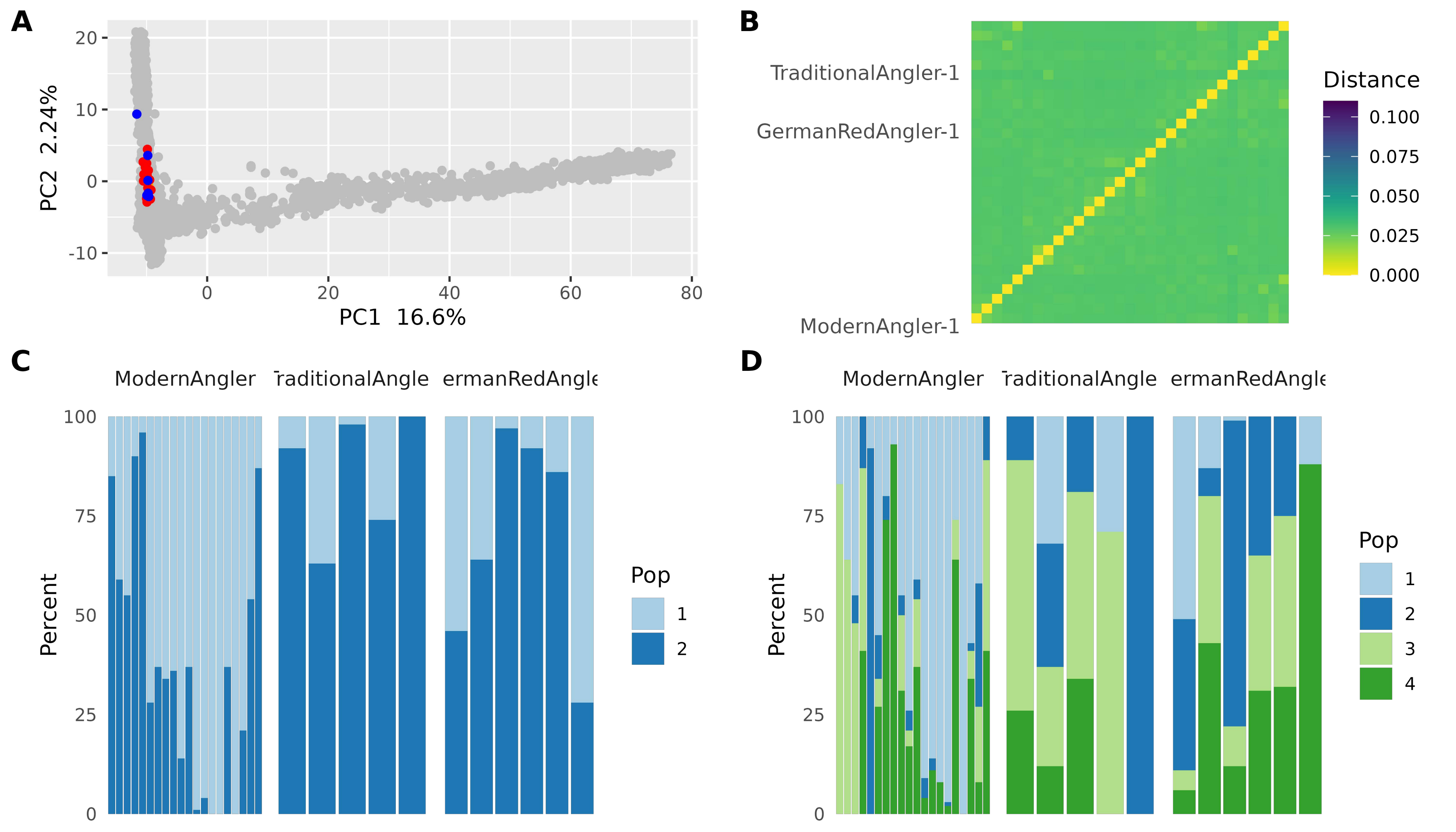

A
PC2 2.24%
PC1 16.6%
20
10
0
-10
0
20
40
60
80
B
TraditionalAngler-1
GermanRedAngler-1
ModernAngler-1
Distance
0.100
0.075
0.050
0.025
0.000
C
ModernAngler
TraditionalAngle
ermanRedAngle
Percent
100
75
50
25
0
Pop
1
2
D
ModernAngler
TraditionalAngle
ermanRedAngle
Percent
100
75
50
25
0
Pop
1
2
3
4

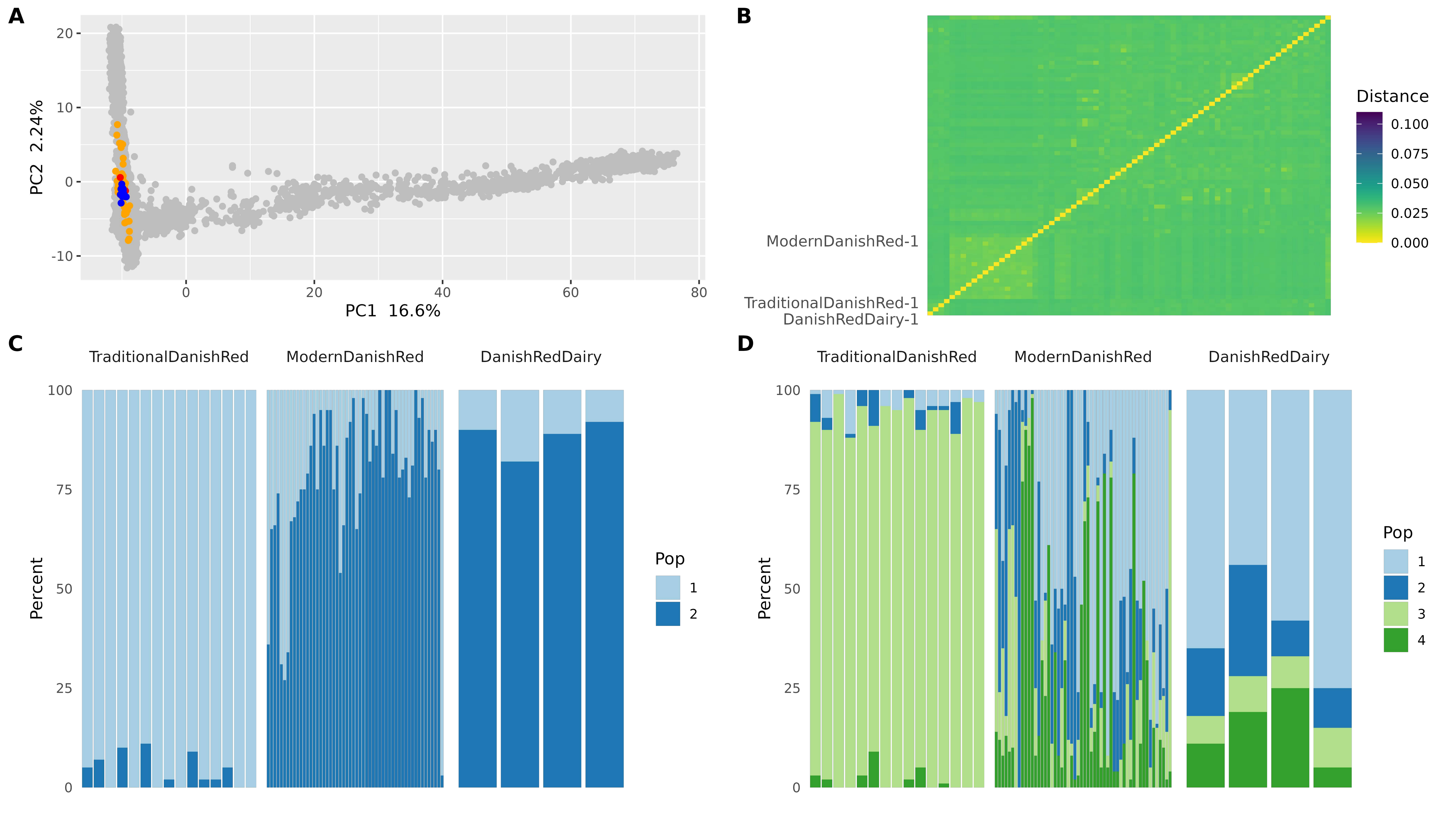

A
B
C
D
PC1 16.6%
PC2 2.24%
ModernDanishRed-1
TraditionalDanishRed-1
DanishRedDairy-1
Distance
0.100
0.075
0.050
0.025
0.000
TraditionalDanishRed
ModernDanishRed
DanishRedDairy
Percent
Pop
1
2
3
4

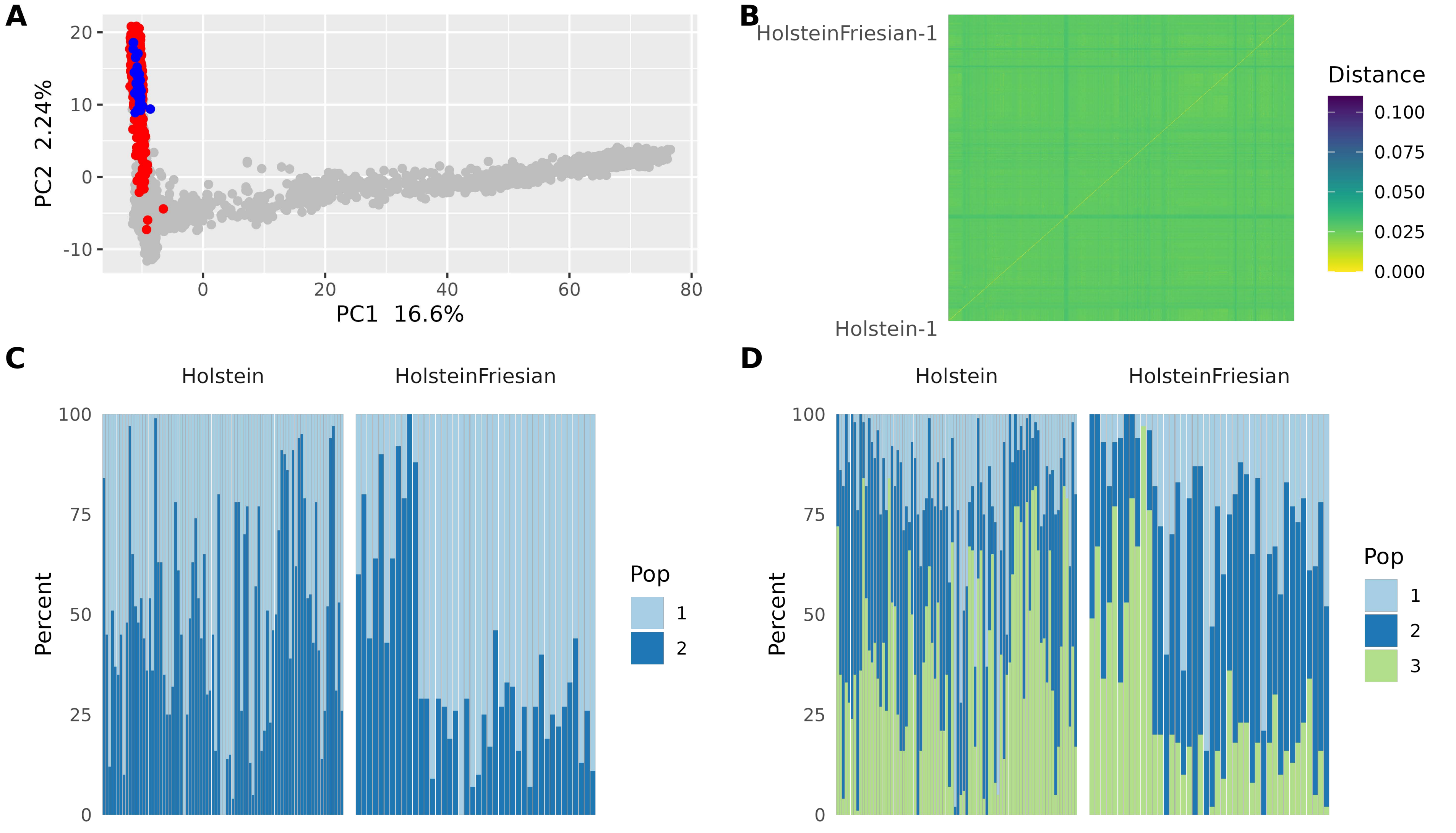

A
B
C
D
PC1 16.6%
PC2 2.24%
HolsteinFriesian-1
Holstein-1
Distance
0.100
0.075
0.050
0.025
0.000
Holstein
HolsteinFriesian
Percent
Pop
1
2
3

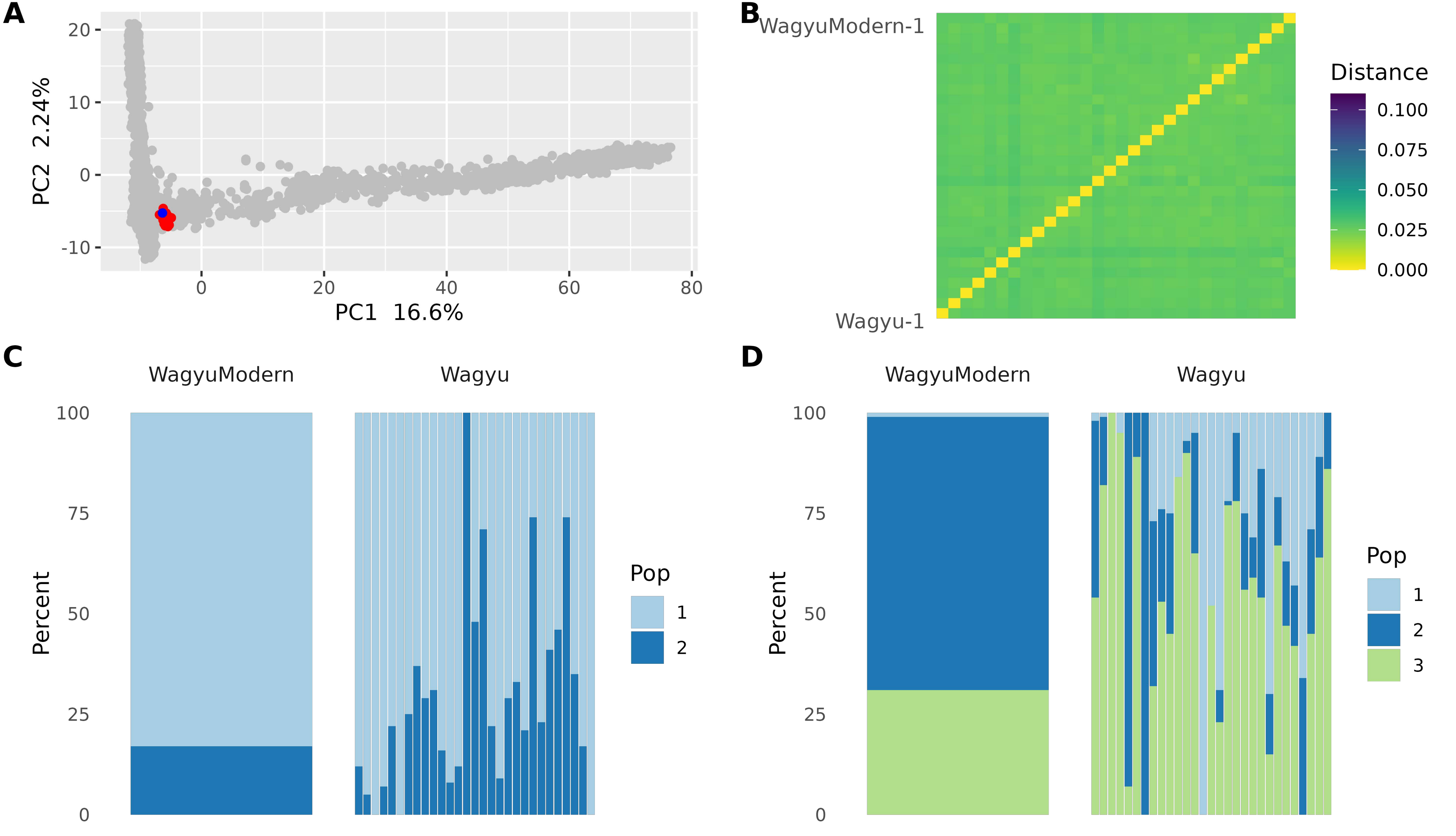
A
B
C
D
PC1 16.6%
PC2 2.24%
WagyuModern-1
Wagyu-1
Distance
0.100
0.075
0.050
0.025
0.000
WagyuModern
Wagyu
Percent
Pop
1
2
3

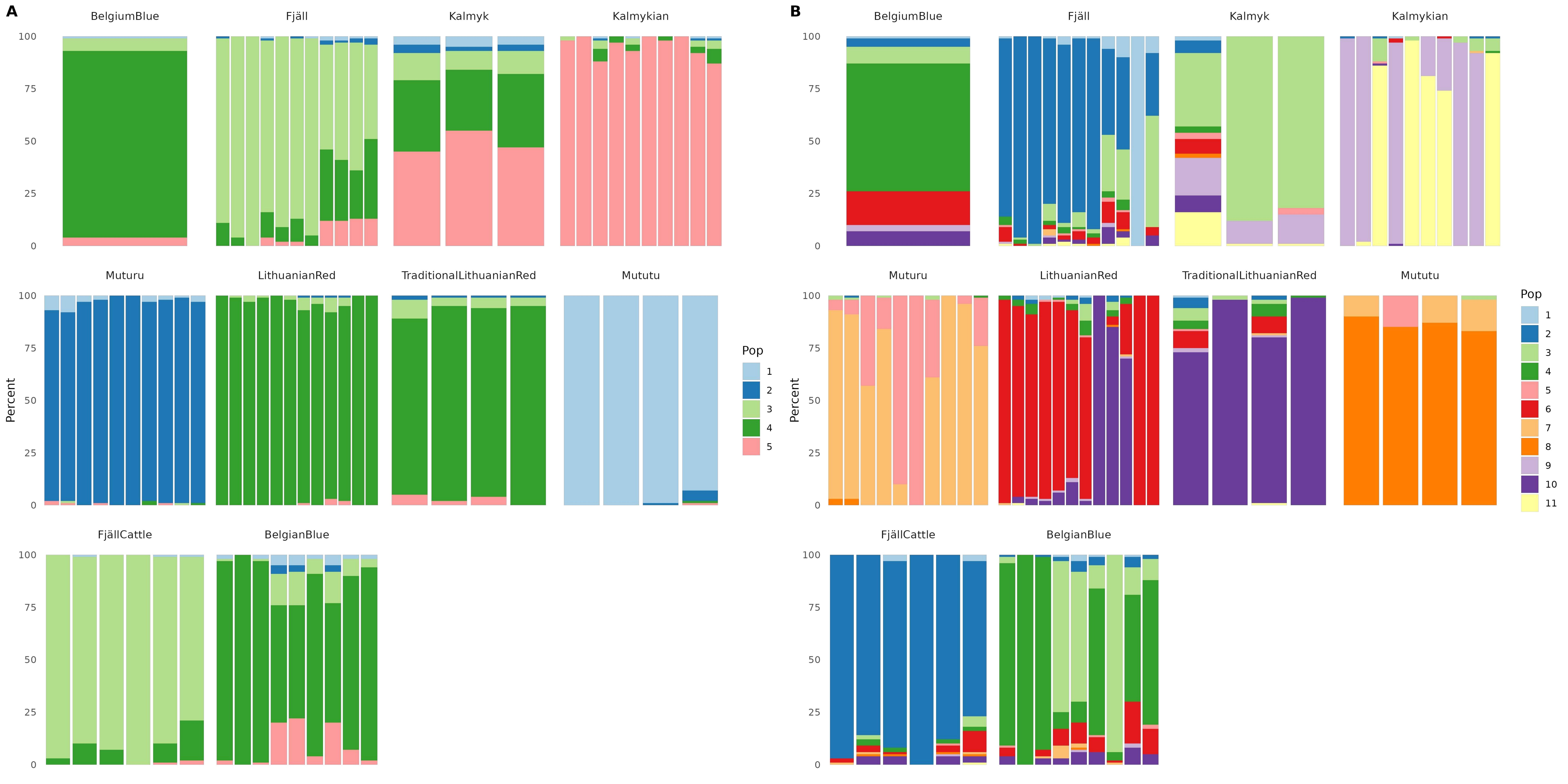

A
B
BelgiumBlue
Fjäll
Kalmyk
Kalmykian
Muturu
LithuanianRed
TraditionalLithuanianRed
Mututu
FjällCattle
BelgianBlue
Percent
Pop
1
2
3
4
5
6
7
8
9
10
11
0
25
50
75
100

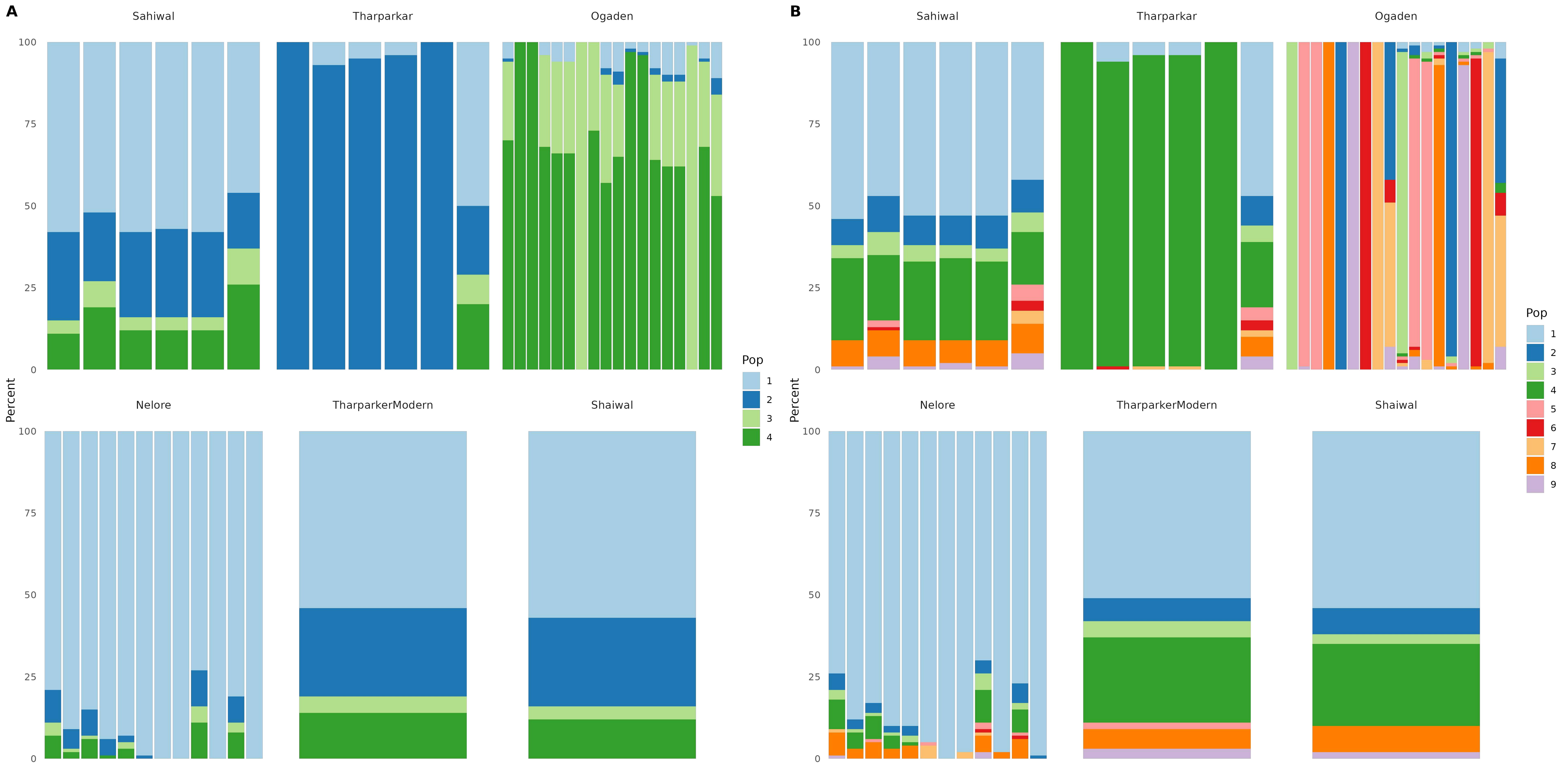

A
B
Sahiwal
Tharparkar
Ogaden
Nelore
TharparkerModern
Shaiwal
Percent
0
25
50
75
100
Pop
1
2
3
4
5
6
7
8
9

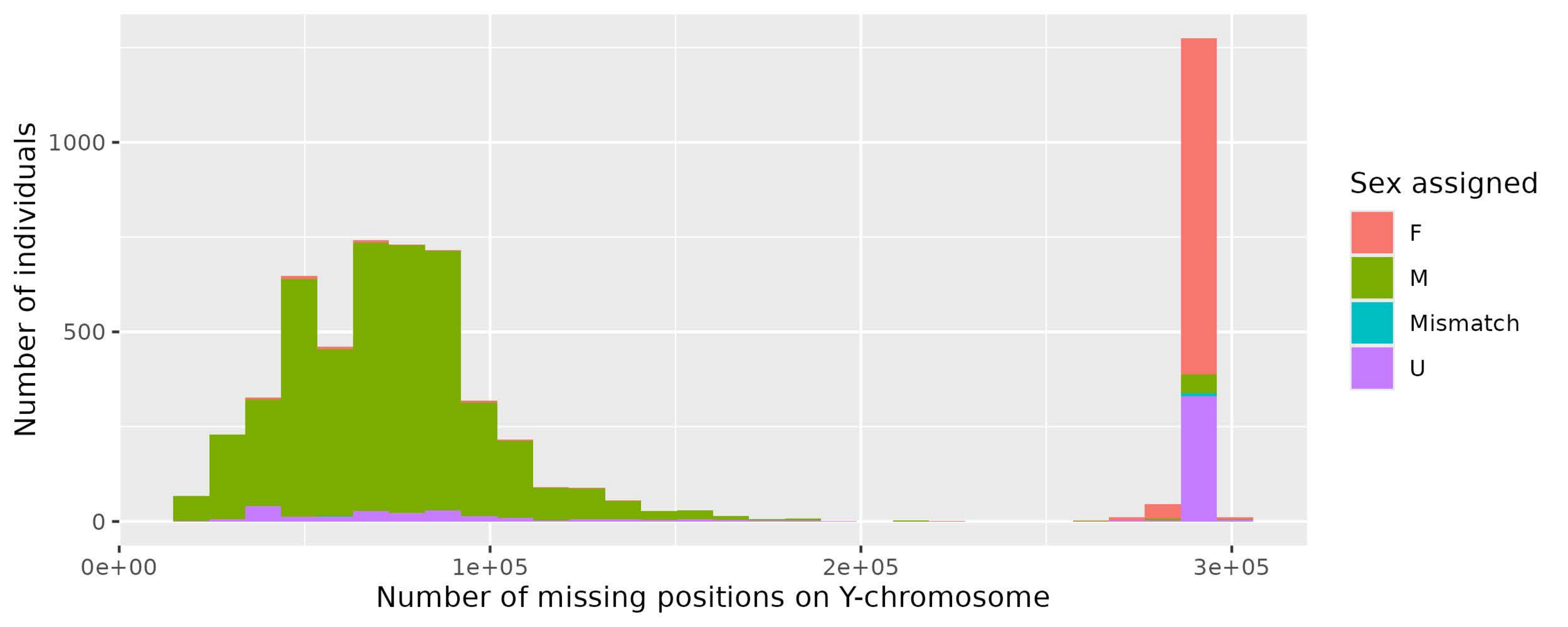

Number of individuals
1000
500
0
0e+00
1e+05
2e+05
3e+05
Number of missing positions on Y-chromosome
Sex assigned
F
M
Mismatch
U

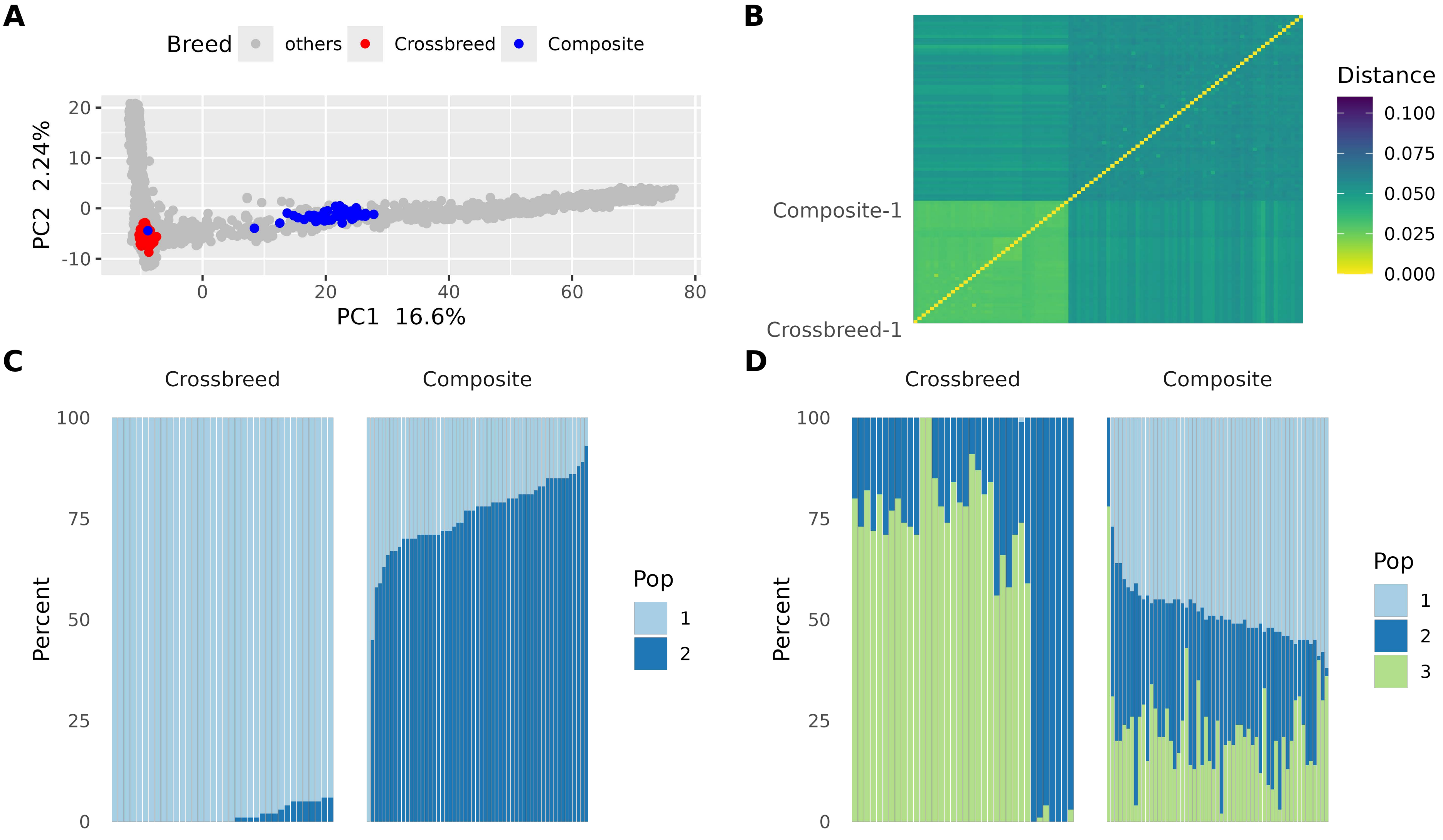
A
Breed
others
Crossbreed
Composite
PC2 2.24%
PC1 16.6%
B
Composite-1
Crossbreed-1
Distance
0.100
0.075
0.050
0.025
0.000
C
Crossbreed
Composite
Percent
Pop
1
2
D
Crossbreed
Composite
Percent
Pop
1
2
3

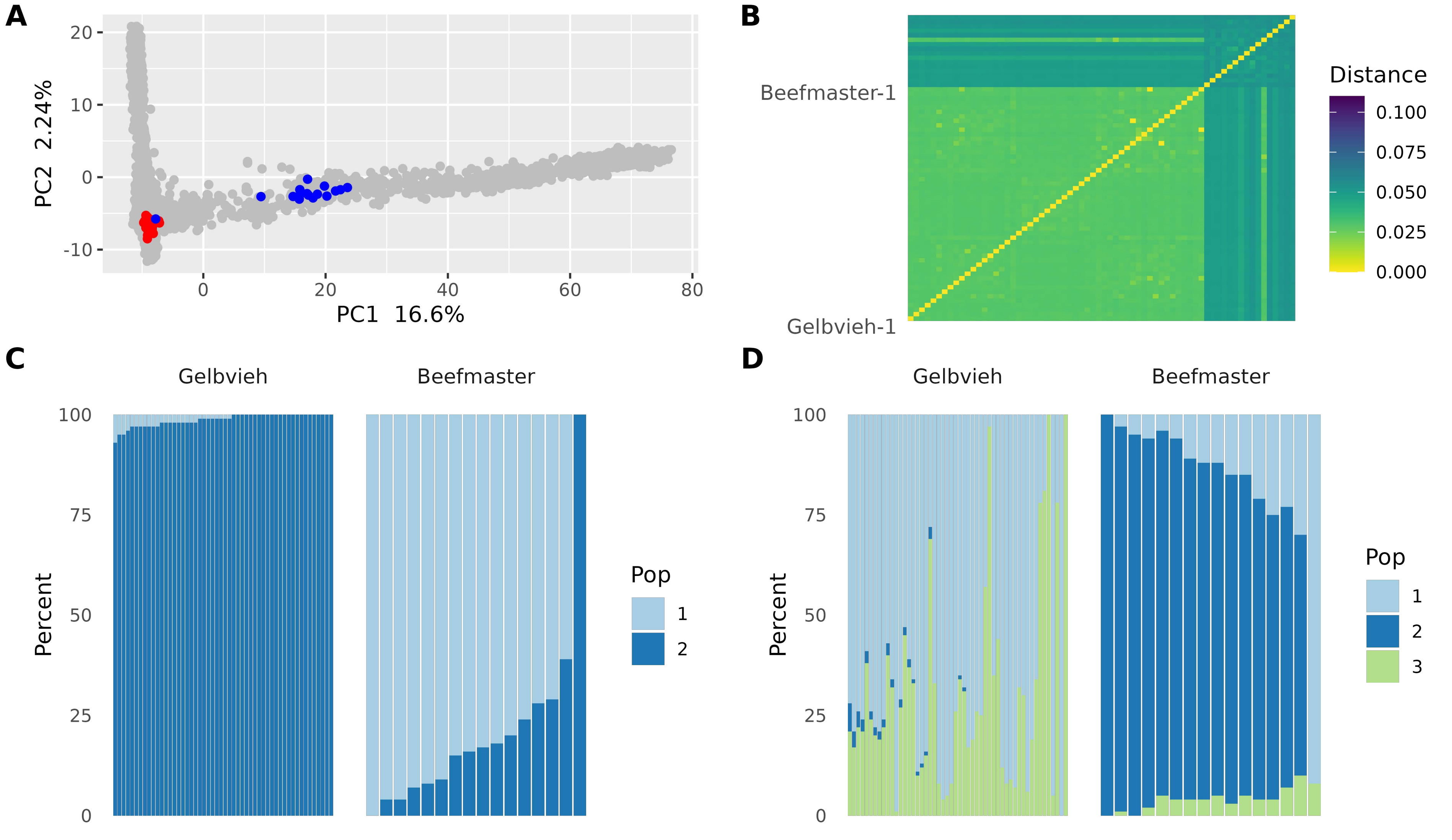
A
PC2 2.24%
PC1 16.6%
B
Beefmaster-1
Gelbvieh-1
Distance
0.100
0.075
0.050
0.025
0.000
C
Gelbvieh
Beefmaster
Percent
Pop
1
2
D
Gelbvieh
Beefmaster
Percent
Pop
1
2
3

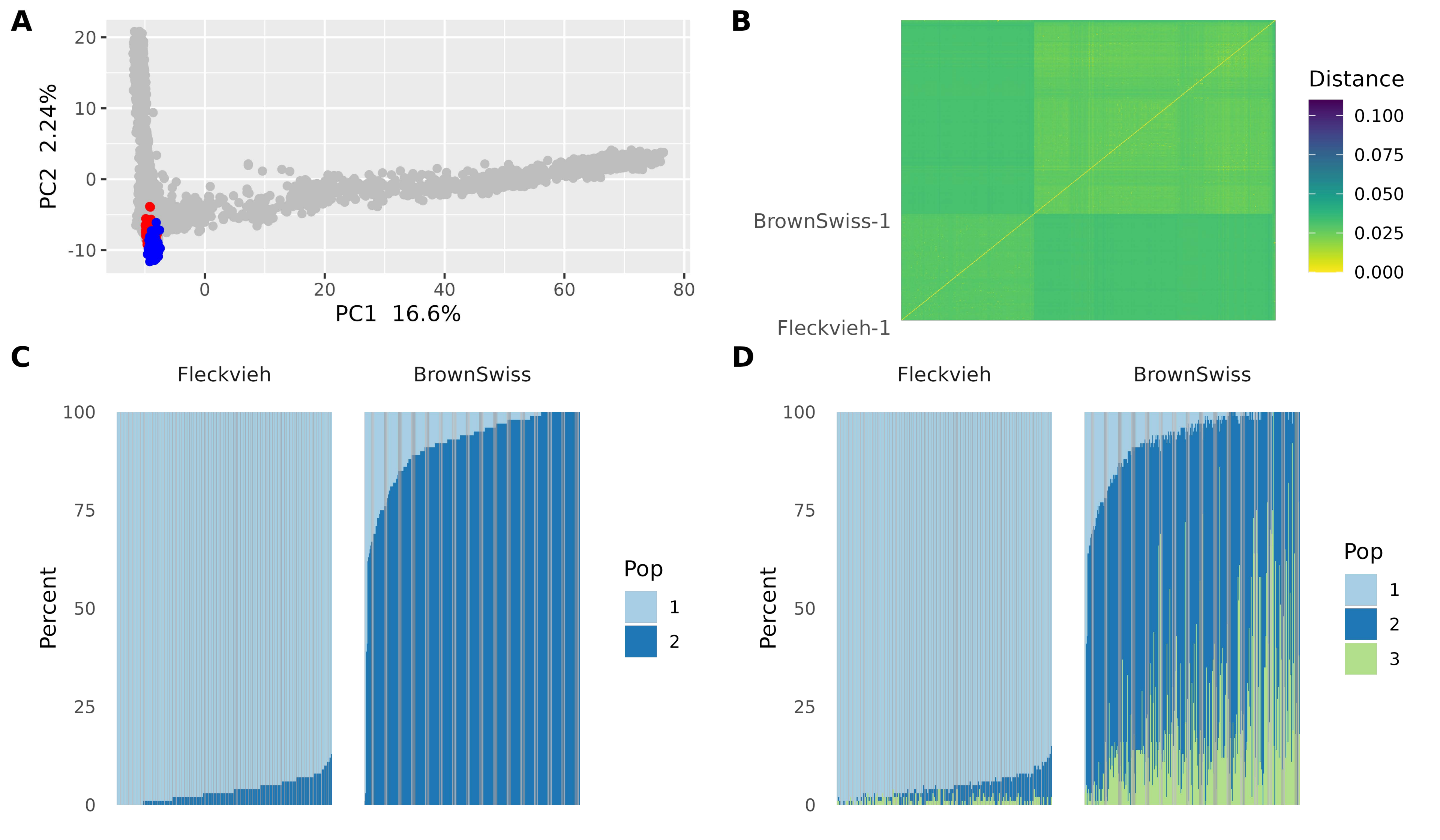

A
PC2 2.24%
PC1 16.6%
20
10
0
-10
0
20
40
60
80
B
BrownSwiss-1
Fleckvieh-1
Distance
0.100
0.075
0.050
0.025
0.000
C
Fleckvieh
BrownSwiss
Percent
100
75
50
25
0
Pop
1
2
D
Fleckvieh
BrownSwiss
Percent
100
75
50
25
0
Pop
1
2
3

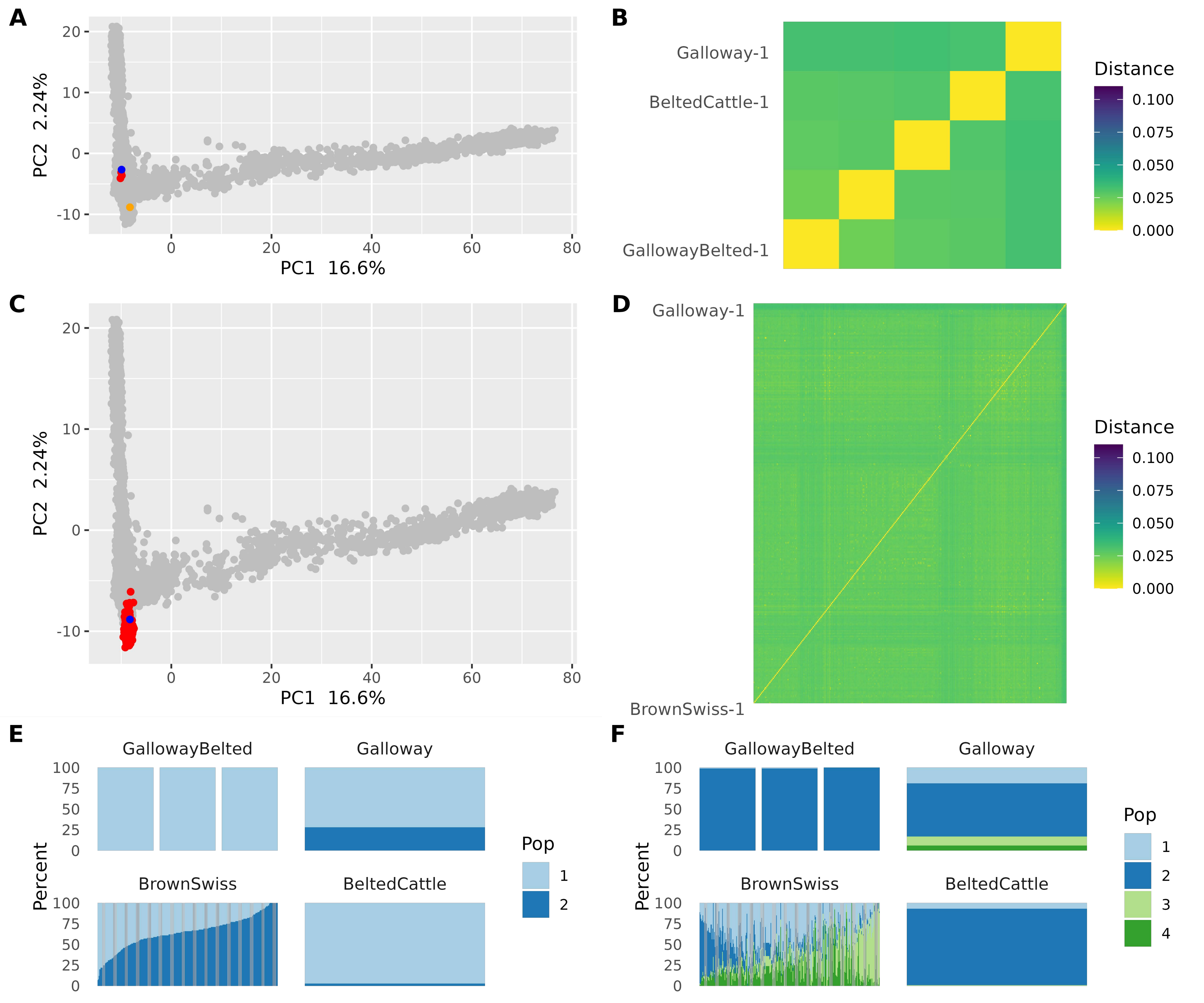
A
PC2 2.24%
PC1 16.6%
B
Galloway-1
BeltedCattle-1
GallowayBelted-1
Distance
C
PC2 2.24%
PC1 16.6%
D
Galloway-1
BrownSwiss-1
Distance
E
GallowayBelted
Galloway
BrownSwiss
BeltedCattle
Percent
Pop
F
GallowayBelted
Galloway
BrownSwiss
BeltedCattle
Percent
Pop

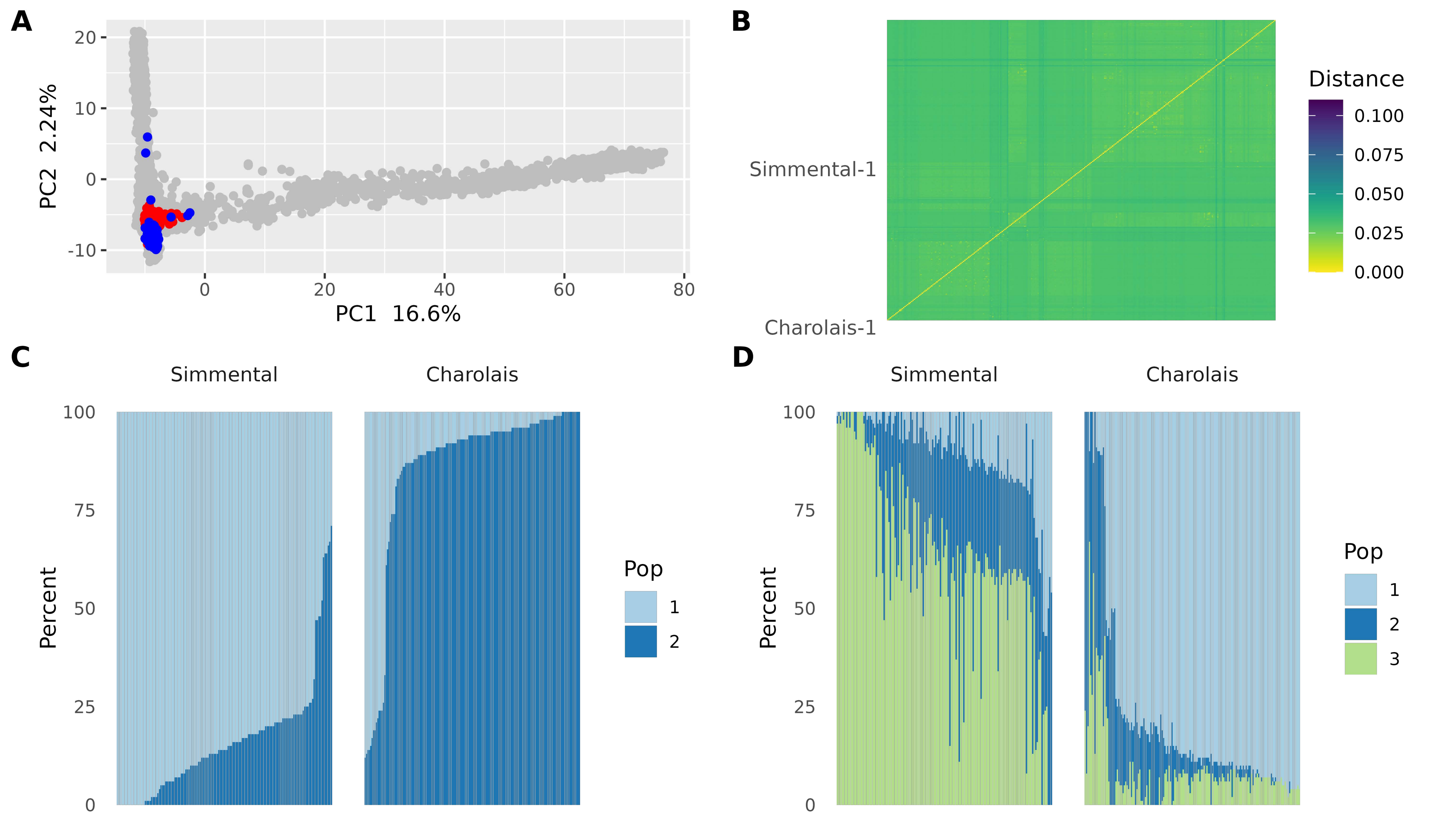
A
B
C
D
PC1 16.6%
PC2 2.24%
Simmental-1
Charolais-1
Distance
0.100
0.075
0.050
0.025
0.000
Simmental
Charolais
Percent
Pop
1
2
3

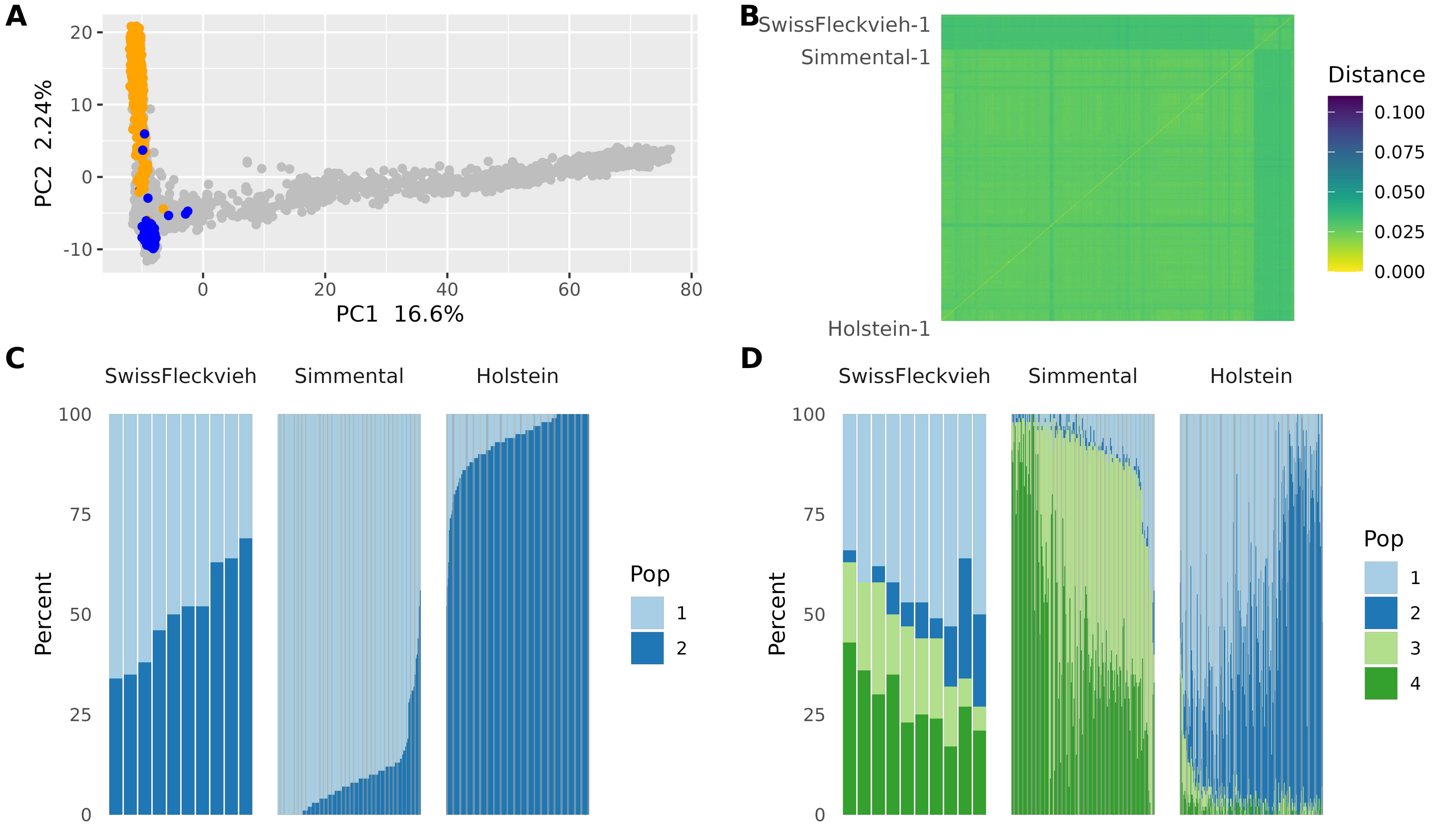
A
PC2 2.24%
PC1 16.6%
B
SwissFleckvieh-1
Simmental-1
Holstein-1
Distance
0.100
0.075
0.050
0.025
0.000
C
SwissFleckvieh
Simmental
Holstein
Percent
Pop
1
2
D
SwissFleckvieh
Simmental
Holstein
Percent
Pop
1
2
3
4

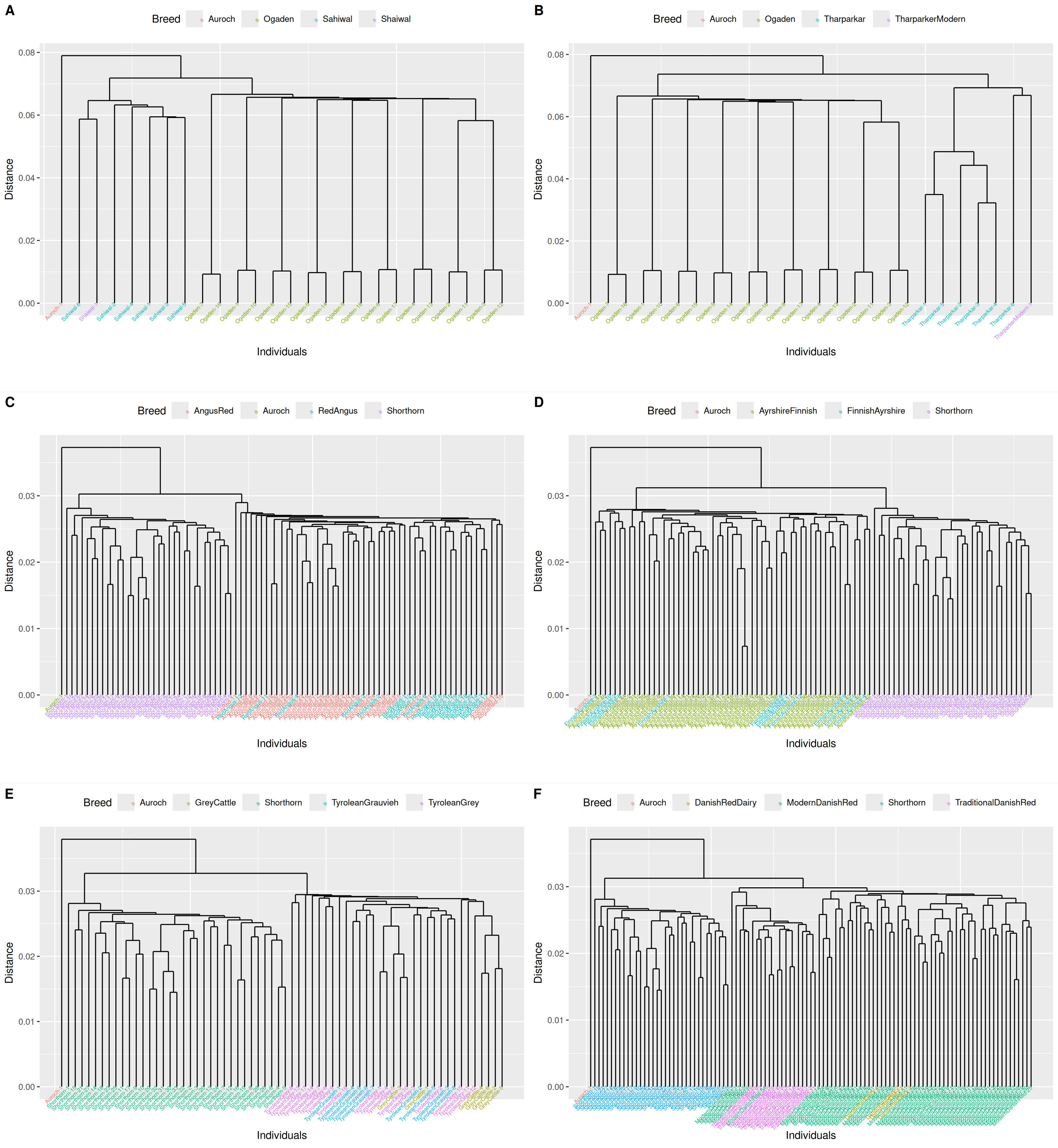

A
Breed
Auroch
Ogaden
Sahiwal
Shaiwal
Distance
Individuals
B
Breed
Auroch
Ogaden
Tharparkar
TharparkerModern
Distance
Individuals
C
Breed
AngusRed
Auroch
RedAngus
Shorthorn
Distance
Individuals
D
Breed
Auroch
AyrshireFinnish
FinnishAyrshire
Shorthorn
Distance
Individuals
E
Breed
Auroch
GreyCattle
Shorthorn
TyroleanGrauvieh
TyroleanGrey
Distance
Individuals
F
Breed
Auroch
DanishRedDairy
ModernDanishRed
Shorthorn
TraditionalDanishRed
Distance
Individuals

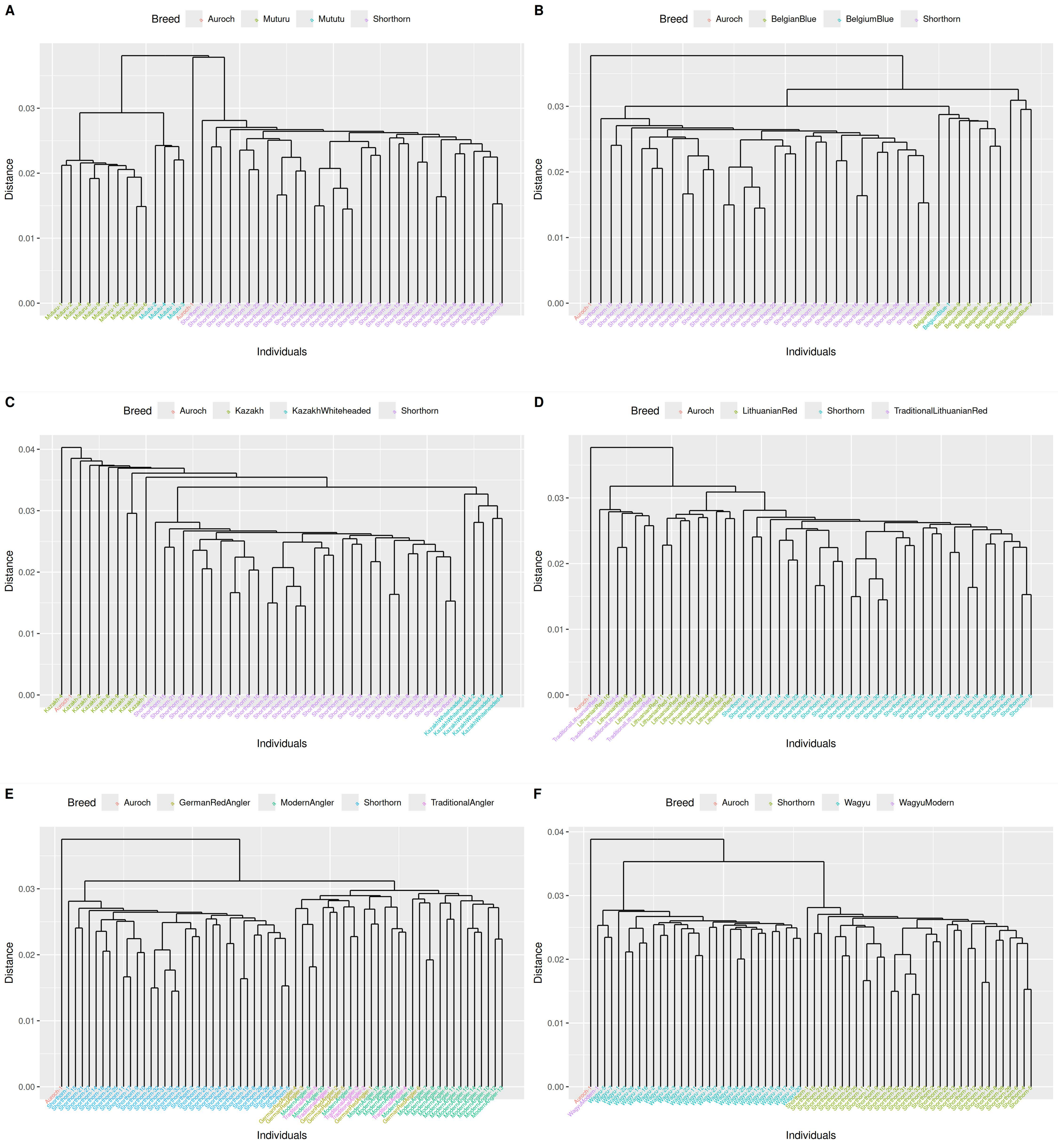
A
Breed
Auroch
Muturu
Mututu
Shorthorn
Distance
Individuals
B
Breed
Auroch
BelgianBlue
BelgiumBlue
Shorthorn
Distance
Individuals
C
Breed
Auroch
Kazakh
KazakhWhiteheaded
Shorthorn
Distance
Individuals
D
Breed
Auroch
LithuanianRed
Shorthorn
TraditionalLithuanianRed
Distance
Individuals
E
Breed
Auroch
GermanRedAngler
ModernAngler
Shorthorn
TraditionalAngler
Distance
Individuals
F
Breed
Auroch
Shorthorn
Wagyu
WagyuModern
Distance
Individuals

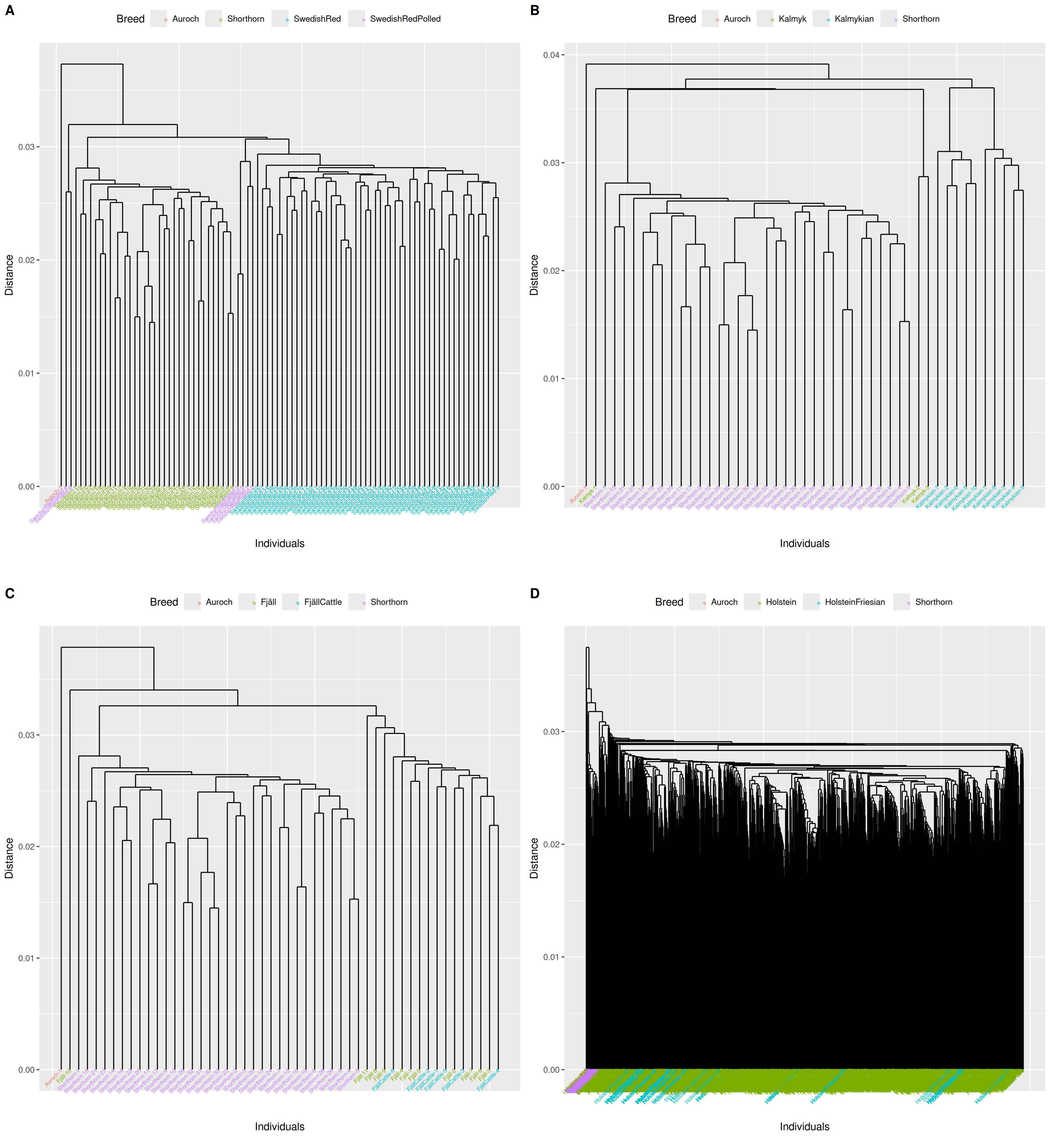

A
Breed
Auroch
Shorthorn
SwedishRed
SwedishRedPolled
Distance
0.00
0.01
0.02
0.03
Individuals
B
Breed
Auroch
Kalmyk
Kalmykian
Shorthorn
Distance
0.00
0.01
0.02
0.03
0.04
Individuals
C
Breed
Auroch
Fjäll
FjällCattle
Shorthorn
Distance
0.00
0.01
0.02
0.03
Individuals
D
Breed
Auroch
Holstein
HolsteinFriesian
Shorthorn
Distance
0.00
0.01
0.02
0.03
Individuals

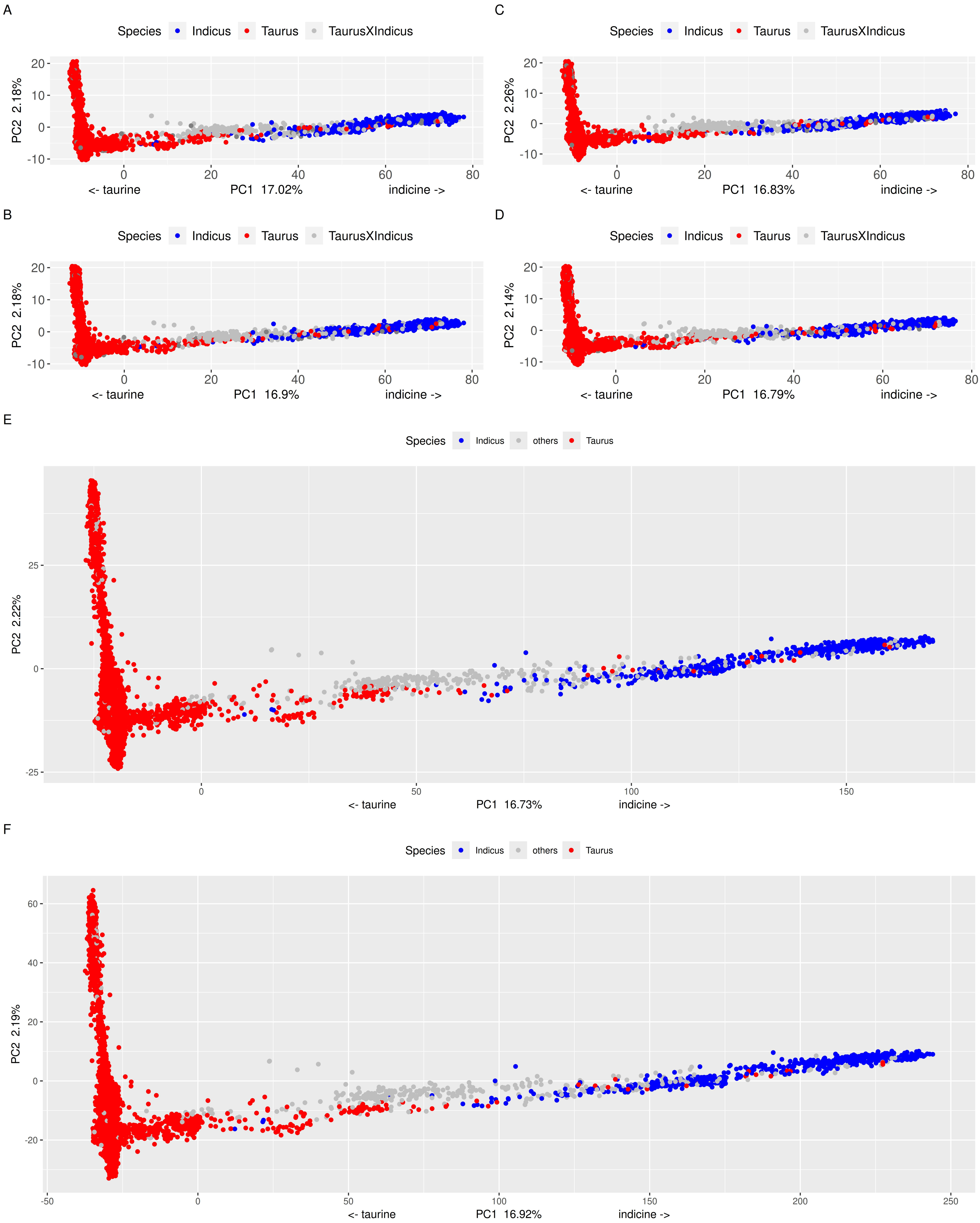
A
Species Indicus Taurus TaurusXIndicus
PC2 2.18%
<- taurine PC1 17.02% indicine ->
C
Species Indicus Taurus TaurusXIndicus
PC2 2.26%
<- taurine PC1 16.83% indicine ->
B
Species Indicus Taurus TaurusXIndicus
PC2 2.18%
<- taurine PC1 16.9% indicine ->
D
Species Indicus Taurus TaurusXIndicus
PC2 2.14%
<- taurine PC1 16.79% indicine ->
E
Species Indicus others Taurus
PC2 2.22%
<- taurine PC1 16.73% indicine ->
F
Species Indicus others Taurus
PC2 2.19%
<- taurine PC1 16.92% indicine ->

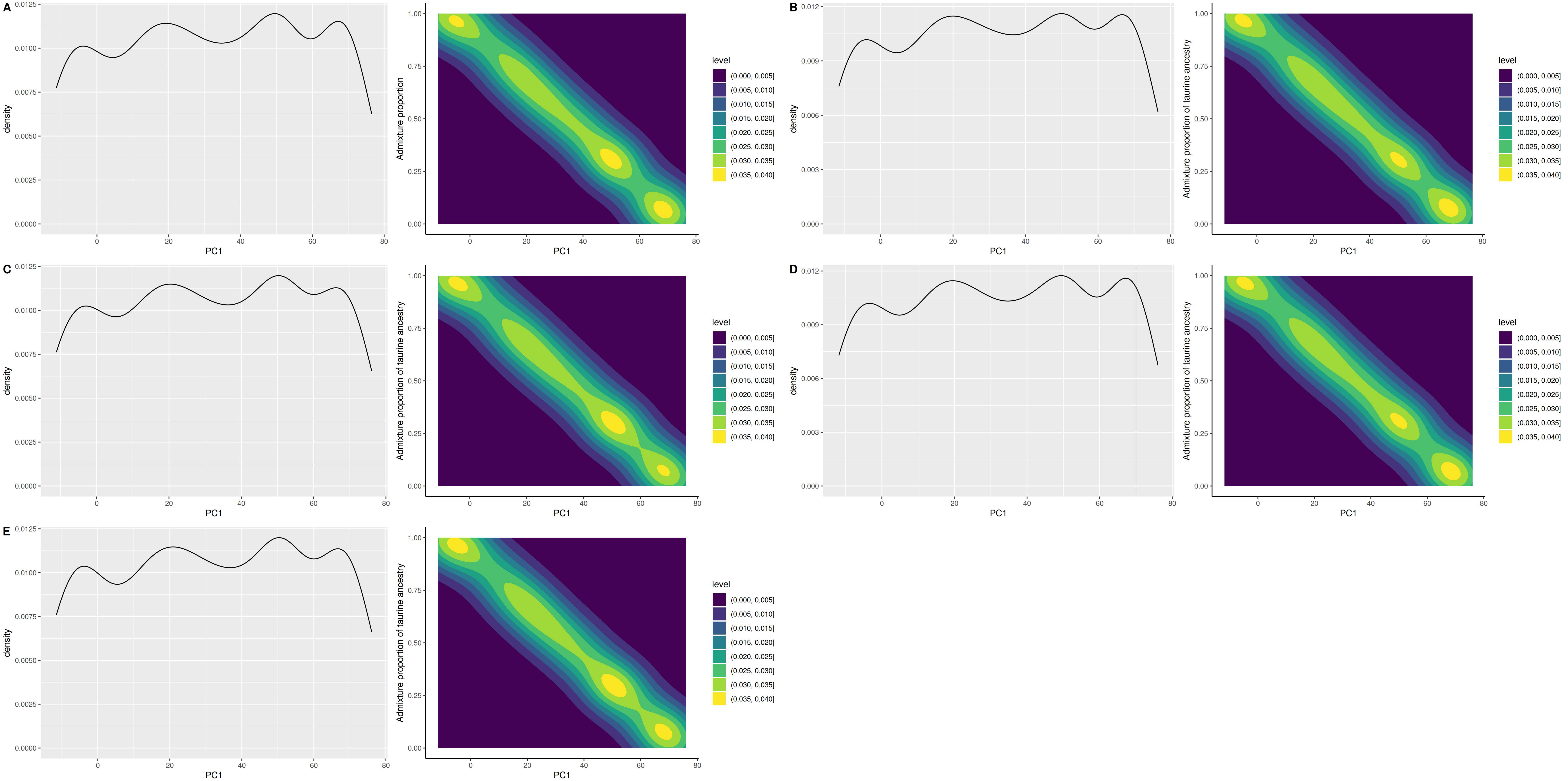

A
B
C
D
E
density
PC1
Admixture proportion
Admixture proportion of taurine ancestry
level
(0.000, 0.005]
(0.005, 0.010]
(0.010, 0.015]
(0.015, 0.020]
(0.020, 0.025]
(0.025, 0.030]
(0.030, 0.035]
(0.035, 0.040]

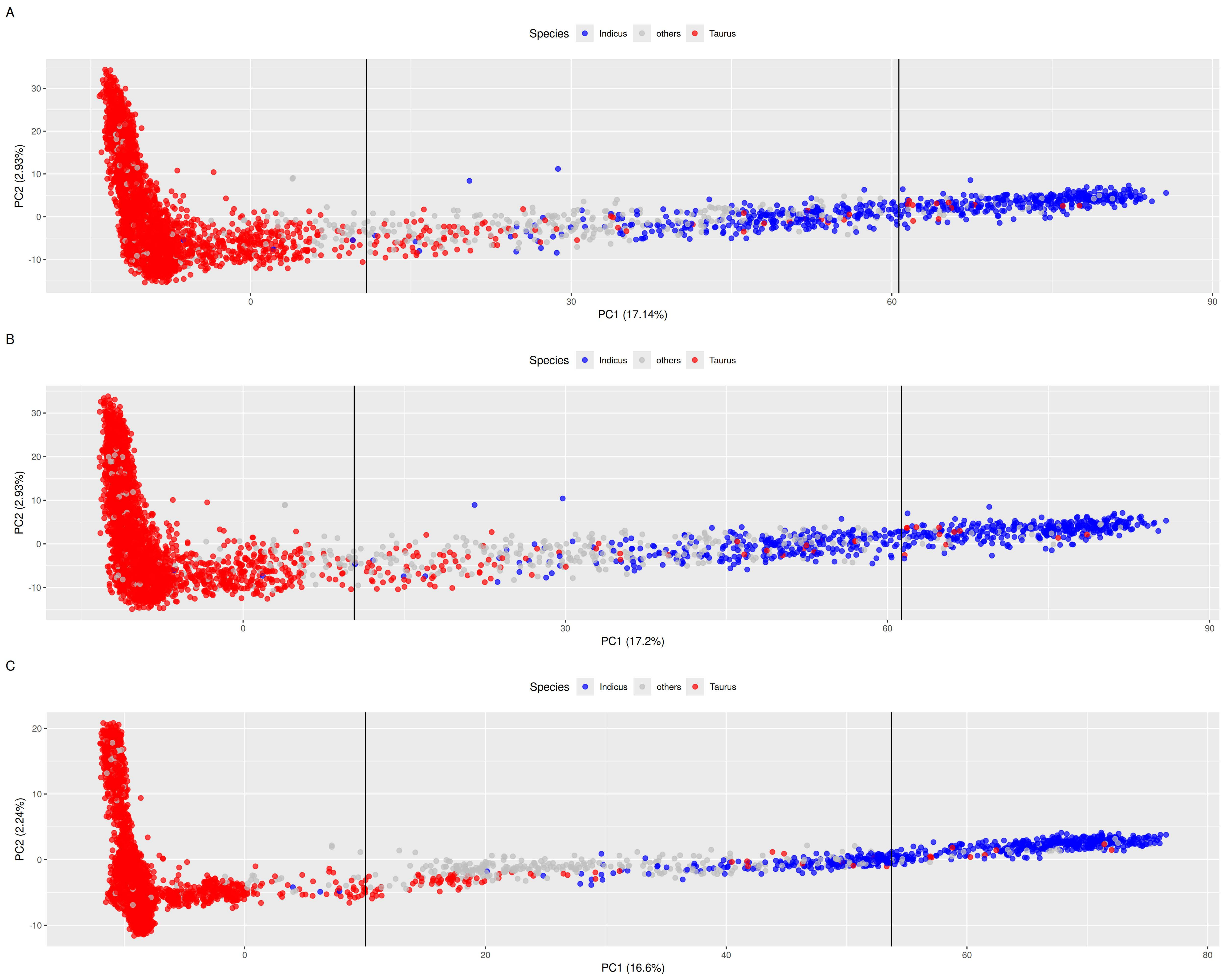
A
Species Indicus others Taurus
PC2 (2.93%)
PC1 (17.14%)
B
Species Indicus others Taurus
PC2 (2.93%)
PC1 (17.2%)
C
Species Indicus others Taurus
PC2 (2.24%)
PC1 (16.6%)
